# Central Nervous System T-cell immune architecture, and not HIV burden, tracks with cognition under long-term viral suppression

**DOI:** 10.1371/journal.ppat.1014351

**Published:** 2026-06-15

**Authors:** Mattia Trunfio, Gemma Caballero, Vanessa Gomez-Moreno, Simon A. Mallal, Celestine N. Wanjalla, Angela Jones, Karen Beeri, Alan Wells, Sarah LaMere, Ben Gouaux, Donald R. Franklin, Michael Corley, Ronald J. Ellis, David J. Moore, Scott L. Letendre, Davey Smith, Antoine Chaillon, Sara Gianella

**Affiliations:** 1 Division of Infectious Diseases and Global Health, Department of Medicine, University of California San Diego, La Jolla, California, United States of America; 2 Department of Clinical and Biological Sciences, University of Turin, Torino, Italy; 3 Department of Medicine, Vanderbilt University Medical Center, Nashville, Tennessee, United States of America; 4 Vanderbilt Technologies for Advanced Genomics, Vanderbilt University Medical Center, Nashville, Tennessee, United States of America; 5 Division of Infectious Diseases, Vanderbilt University Medical Center, Nashville, Tennessee, United States of America; 6 Veterans Affairs Tennessee Valley Healthcare System, Nashville, Tennessee, United States of America; 7 Department of Psychiatry, University of California San Diego, San Diego, California, United States of America; 8 Division of Geriatrics and Palliative Care, Department of Medicine, University of California San Diego, La Jolla, California, United States of America; NIH, NIAID, UNITED STATES OF AMERICA

## Abstract

Despite effective antiretroviral therapy, HIV persists in the central nervous system (CNS) and may contribute to neuroinflammation and cognitive impairment. How viral persistence, immune responses, and regional CNS T-cell architecture relate to cognitive functioning remains unclear. We performed a cross-sectional, multi-compartmental immune-genomic study in 12 people with HIV on long-term viral suppression enrolled in the Last Gift rapid autopsy program. Quantitative HIV reservoir measures (total-episomal DNA, unspliced-multiply spliced RNA) and paired αβ T-cell receptor repertoire (TCRR) sequencing were performed in peripheral blood mononuclear cells and five CNS regions: hippocampus, frontal motor cortex, basal ganglia, occipital cortex, and spinal cord. Cognitive performance was assessed within one year of death. Tissue-resolved associations between cognition and HIV reservoir, TCRR architecture (richness, diversity, clonality), and pathogen-specific T-cell clonotypes (HIV, CMV, EBV, and riboflavin derivatives) were evaluated using participant-clustered multivariable models. False discovery rate was applied. HIV DNA and RNA were detectable across all tissues but were not associated with cognitive performance or TCRR metrics. Peripheral TCRR architecture was unrelated to cognition, whereas higher TCRR richness and diversity in the hippocampus and spinal cord were associated with worse verbal, motor, and attention/working memory scores. Higher TCRR richness in the spinal cord was also associated with better recall. T-cell receptor clonotype frequency distributions differed across CNS regions, consistent with regional immune compartmentalization. Epitope-inference analyses revealed pathogen-dependent associations: higher number of HIV–specific T-cell clonotypes in the basal ganglia was associated with better global and attention/working memory scores, whereas riboflavin derivative–specific clonotypes in frontal motor cortex were associated with better motor performance. CMV-specific clonotypes showed nominal associations with worse learning and memory. CNS–localized T-cell receptor architecture and antigenic imprinting related more closely to neurocognitive variability than quantitative measures of HIV persistence under viral suppression, highlighting regional specialization of T-cell responses as a potential correlate of brain health.

## Author summary

Even when antiretroviral therapy successfully suppresses HIV in the blood, the virus persists in tissues, including the brain and spinal cord. Many people living with HIV continue to experience cognitive difficulties despite long-term treatment, but the biological reasons remain unclear. We asked whether cognitive performance was more closely related to the amount and activity of HIV in the central nervous system or to characteristics of local T cells. Using tissues donated through a rapid autopsy program, we measured viral persistence and profiled the diversity and composition of T-cell receptors across multiple brain regions and in blood. We found that the quantity of viral DNA and RNA in brain tissues was not associated with cognitive performance. In contrast, features of the T-cell receptor repertoire within specific brain regions were linked to differences in memory, attention, and motor function. These patterns varied across regions, indicating that immune architecture differs within the brain. Our findings suggest that regional T-cell organization in the central nervous system, rather than the amount of persistent virus alone, relates to long-term brain health in people living with HIV. Understanding these immune dynamics may help guide future strategies to preserve cognitive function.

## 1. Introduction

Despite the success of antiretroviral therapy (ART), HIV persists within the central nervous system (CNS), contributing to chronic neuroinflammation and neurocognitive impairment. [1] Proviral DNA remains detectable in CNS tissues during long-term viral suppression, with variable levels and transcriptional activity across regions. [2,3] Chronic immune activation and neuroinflammation can sustain HIV persistence [4] and ongoing HIV transcription and protein expression, even in the absence of productive viral replication, may exert neurotoxic effects. [5] Together, these processes may establish a self-perpetuating cycle of neuroinflammation under ART, ultimately affecting brain and mental health of people with HIV (PWH). Consistent with this, HIV-associated neurocognitive impairment remains prevalent in the ART era and continues to impact the quality of life and healthy aging of PWH. [6] However, the underlying exact mechanisms remain incompletely understood. In particular, the relative contribution of HIV persistence as opposed to co-infections and comorbidities remains unclear. [7,8] This limited understanding of the mechanisms linking these processes to neuroinflammation and the consequences and localization of HIV persistence within the CNS constrain eradication strategies and interventions for CNS complications.

Restricted access to CNS tissues from living individuals remains a major challenge. Much of the field relies on *in vitro* or animal models, or extrapolations from blood and CSF, which may not accurately reflect CNS biology. [9–11] Human data are scarce. A recent post-mortem study found no difference in intact proviral burden in frontal white matter between PWH with and without cognitive impairment. [12] However, intact proviral levels correlated with neuroinflammatory gene expression, suggesting that transcriptionally active reservoirs may contribute to CNS injury. [12] Adding further complexity, CNS immune responses exhibit pronounced regional specificity, [13–15] which can influence heterogeneous reservoir persistence and activity, [16] as well as downstream neuroinflammation, underscoring the need for region-resolved human studies. However, most human studies rely on soluble or transcriptomic measurements that capture relatively proximal and dynamic immune states and may not fully reflect the cumulative, compartmentalized immune architecture shaped by long-term and tissue-specific processes.

The T cell receptor repertoire (TCRR), defined as the ensemble of all T cell receptors within an individual, has emerged as an important correlate of neuroimmune pathology in several non-HIV conditions, including multiple sclerosis, neurodegenerative disorders, and psychiatric conditions. [17–19] In these, perturbations in TCRR architecture, such as oligoclonality, skewed repertoire distribution, and reduced richness and diversity in blood and CSF have been linked to neuroinflammation, cognitive impairment, disease progression, and neuro-behavioral traits. [17–22] Furthermore, longstanding hypotheses implicating chronic viral infections such as EBV and HSV-1 in neurological disorders are being recently revisited in light of evidence that these pathogens can imprint lasting alterations in the TCRR. [17,23,24] Altogether, TCRR architecture may represent a mechanistic interface between chronic antigenic exposure and neurocognitive dysfunction, capturing a temporally integrated dimension of neuroimmune activity that is complementary to conventional inflammatory readouts.

A functional, rich, and diverse TCRR is essential for preventing autoimmunity and mounting effective immune responses. [25] Within the CNS, homeostatic TCRR shapes the local immune milieu, supporting neuronal and glial function, while modulating immune surveillance and restraining dysregulated inflammation. [17,22,26,27] In animal models, homeostatic TCRR supports neurogenesis, synaptic plasticity, and cognition: e.g., a diverse and rich TCRR promotes IL-4-mediated modulation of hippocampal neurogenesis and microglia neurotrophic responses, thereby enhancing learning and memory. [22,27] Therefore, chronic alterations in TCRR architecture could plausibly contribute to neuro-injury, and ultimately to poorer cognition.

In PWH, TCRR perturbations arise early and persist despite effective viral suppression. [28–31] Off ART, high viral loads are associated with reduced repertoire diversity, oligoclonality, and other TCRR abnormalities in peripheral blood. [28–31] Although ART decreases HIV-specific clonal expansions, persistent TCRR perturbations remain, and correlate with immune activation and T-cell exhaustion. [30] Despite this, the contribution of TCRR alterations to the brain and mental health of PWH remains largely unexplored, data on TCRR perturbations in tissues other than blood are scarce, [32] and no prior studies have examined how the regional CNS TCRR architecture and HIV reservoir jointly relate to cognition.

To address this gap, we integrated quantitative HIV reservoir measurements with paired TCRR profiling across five CNS regions and peripheral blood from well-characterized PWH on ART in the Last Gift study, a rapid autopsy program, [33] and the and California NeuroHIV Tissue Network (CNTN) at the University of California San Diego (UCSD). We investigated two biologically plausible mechanisms, regional HIV persistence and TCRR architecture, whose relative association to cognition under suppressive ART remains unknown. We hypothesized that a larger and more transcriptionally active HIV reservoir in the CNS would associate with poorer cognitive performance, and that lower TCRR diversity and richness, alongside higher clonality, would similarly relate to worse cognitive performance. Lastly, we explored whether the abundance of T cell clonotypes specific to HIV, EBV, CMV, and bacterial riboflavin-derived antigens contributed to inter-individual cognitive variability.

## 2. Results

### 2.1. Study population

Twelve participants from the Last Gift/CNTN cohort met the inclusion criteria and underwent cognitive assessment within one year before autopsy (median interval 8 [4–10] months). Participants were predominantly white (83.3%), male at birth (83.3%), with a mean age of 65 ± 8 years, and median duration of HIV and viral suppression on ART of 23 (20–29) and 18 (13–23) years. Median last and nadir CD4^+^ T-cell counts were 247 (133–376) and 130 (59–164) cells/μL. All participants with available data of CD8 + T-cells (n = 7) had a CD4/CD8 ratio <1, consistent with persistent immune dysregulation despite long-term viral suppression: median CD8^+^ T-cell count and CD4/CD8 ratio were 278 (209–366) cells/μL and 0.7 (0.6-0.9). Causes of death included cancer (75.0%, none involving the CNS) and end-stage organ disease (25.0%). Three quarters had cognitive impairment, with variability in both the severity of global impairment (among impaired, median global deficit score, GDS, of 0.8, min-max 0.6-1.6) and of individual domains involved (e.g., attention/working memory and executive functioning were impaired in 25.0% and 33.3%, vs learning and motor functioning in 50.0% and 75.0%). Prior cognitive assessment was available for 8 participants, performed at a median of 20 (15–33) months before the assessment used in this study. Among these, the median change in GDS between the prior and final assessment was 0.28 (−0.06 to 0.53). Two participants transitioned from normal to impaired performance, whereas the remaining participants stayed within the same category, including two who improved GDS staying below the 0.5 GDS cut-off, and four persistently impaired with worsening GDS. After normalizing for time between assessments, the median change in GDS was + 0.01/month (IQR −0.004 to +0.02), corresponding to approximately +0.12/year. Thus, although some quantitative worsening occurred, global impairment status was largely stable over the available pre-autopsy interval.

### 2.2. TCRR architecture and HIV reservoir across peripheral blood and the CNS

**Fig 1:**
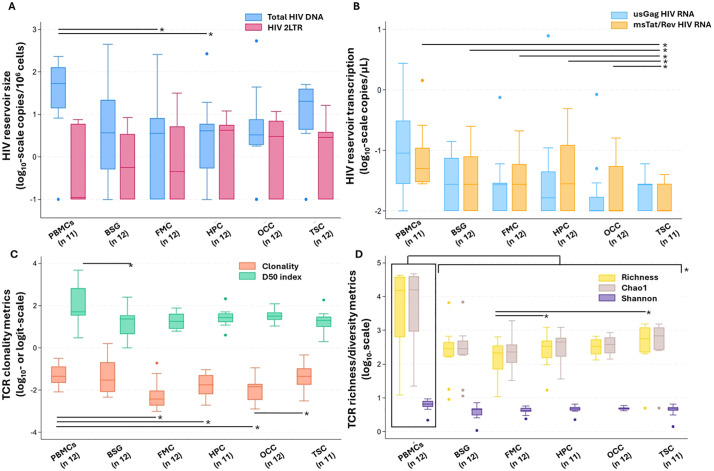
HIV reservoir and TCR characteristics in peripheral blood and the CNS. Top: boxplots show total HIV DNA and 2LTR **(A)**, and usGag RNA and msTat/Rev RNA levels in tissues **(B)**; all values were log_10_-scaled. Bottom: boxplots show TCRR clonality and D50 index (C; logit- and log_10_-scaled, respectively), and TCRR richness and diversity metrics across tissues (D; log_10_-scaled). Box boundaries represent the first and third quartiles, center lines represent medians. Only significant differences at pairwise comparisons of estimates between tissues from linear mixed-effects models are shown (*Benjamini-Hochberg FDR-adjusted q values<0.05; panel D shows significant differences between all three metrics in PBMCs vs all CNS regions). Models for HIV DNA controlled for age, cause of death, last CD4 + T cell count, duration of HIV, and ART regimen, as those for HIV RNA that also controlled for total HIV DNA. Models for TCR metrics controlled for age, cause of death, last CD4 + T cell count, duration of HIV, and sequencing depth. Abbreviations: FMC, frontal motor cortex; BSG, basal ganglia; OCC, occipital cortex; HPC, hippocampus; TSC, thoracic spinal cord; PBMCs, peripheral blood mononuclear cells; FDR, False Discovery Rate; TCRR, T-cell receptor repertoire.

**Fig 2:**
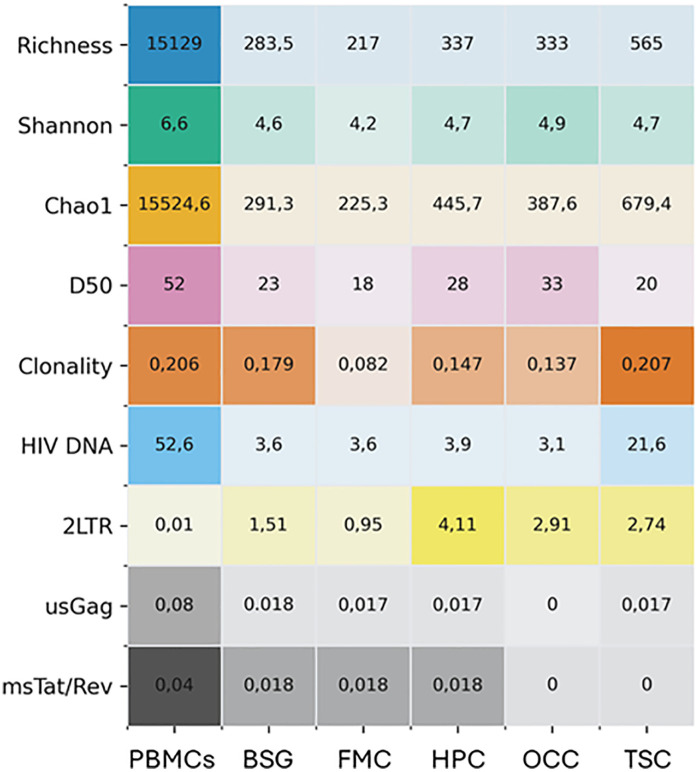
Distribution of HIV reservoir and T-cell receptor repertoire metrics across tissues. Heatmap showing HIV reservoir measurements and TCRR metrics across anatomical sites in the study population. Columns represent tissues, and rows represent individual metrics. Each metric is displayed using an independent color scale normalized within that metric, with lighter shades indicating lower values and darker shades indicating higher values. Numeric values shown in each cell correspond to the original median value. Abbreviations: 2LTR, HIV Two-Long Terminal Repeat DNA; usGag, unspliced HIV Gag RNA; msTat/Rev, multiply spliced HIV Tat/Rev RNA; PBMCs, peripheral blood mononuclear cells; BSG, basal ganglia; FMC, frontal motor cortex; HPC, hippocampus; OCC, occipital cortex; TSC, thoracic spinal cord; TCRR, T-cell receptor repertoire.

The HIV reservoir across tissues is shown in Figs 1A-1B and 2 (details in S1 Table). Total HIV DNA levels were higher in peripheral blood mononuclear cells (PBMCs) compared to all CNS regions (all p < 0.05), except thoracic spinal cord (TSC; p = 0.069). After false discovery rate (FDR) correction, HIV DNA levels in PBMCs remained significantly higher than in frontal motor cortex (FMC) and hippocampus (HPC; Fig 1A). No tissue-specific differences were detected in episomal HIV DNA or unspliced Gag RNA (_us_Gag) levels (Fig 1A-1B). In contrast, multiply spliced Tat/Rev RNA (_ms_Tat/Rev) levels were significantly lower in the TSC than in all other CNS regions and PBMCs (all q < 0.05; Fig 1B). In all CNS regions, total HIV DNA correlated with _us_Gag but not with _ms_Tat/Rev RNA nor with 2LTR DNA, and no consistent relationships were observed between early and late transcripts (S2 Table).

TCRR metrics across tissues are shown in Figs 1C-1D and 2 (details in S1 Table). Diversity and richness in PBMCs were significantly higher than those in all CNS regions (for all metrics and pairwise comparisons q < 0.05, except q = 0.065 for Shannon index in PBMCs vs occipital cortex, OCC). Within the CNS, TCRR diversity and richness were largely similar across regions (Fig 1D), except for higher richness in TSC and HPC compared to FMC (q = 0.007 and q = 0.013). PBMCs showed significantly higher clonality compared to FMC (q = 0.015), HPC (q = 0.022), and OCC (q = 0.015), while within the CNS, TSC displayed higher clonality than OCC (q = 0.022; Fig 1C). A significantly larger number of clonotypes contributed to half of the repertoire in PBMCs compared to FMC (D50 index; q = 0.0015; Fig 1C).

**Fig 3:**
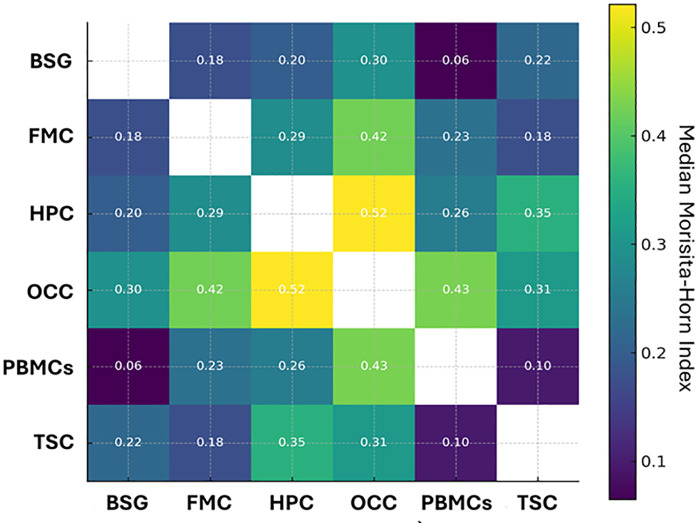
Median tissue–tissue overlap of the TCRR quantified using the Morisita–Horn index. Heatmap showing pairwise median Morisita-Horn index across all six tissues for all participants. The index measures similarity in TCR clone frequency distributions between tissues; higher values indicate greater overlap. Values within cells represent median indices across participants. Abbreviations: FMC, frontal motor cortex; BSG, basal ganglia; OCC, occipital cortex; HPC, hippocampus; TSC, thoracic spinal cord; PBMCs, peripheral blood mononuclear cells; TCR, T-cell receptor.

Despite similar richness and diversity across CNS, the overlap in the frequency distributions of TCRR sequences (Morisita index) was low-to-moderate (Fig 3). The highest overlap was between OCC and HPC (median Morisita index 0.52 [0.31-0.62]), indicating that approximately half of the clonotype frequency distribution was shared between these two regions. Moderate overlap was detected between OCC and all other regions (vs PBMCs: 0.43 [0.21-0.62]; FMC: 0.42 [0.19-0.60]; TSC: 0.31 [0.16-0.62]; basal ganglia, BSG: 0.30 [0.04-0.49]), and between HPC and TSC (0.35 [0.24-0.65]). All remaining tissue pairs showed weak overlap, highlighting substantial regional specificity in TCRR sequence composition despite similar aggregate diversity and richness.

Globally and across individual regions, TCRR clonality was not associated with HIV DNA levels. However, when considering transcriptional activity, higher levels of _us_Gag RNA (q = 0.005) and _ms_Tat/Rev RNA (q = 0.025) in the HPC and BSG, respectively, were associated with higher TCRR clonality. TCRR diversity and richness were not associated with HIV reservoir metrics in any region.

### 2.3. TCRR diversity and richness in hippocampus and spinal cord are associated with cognitive functioning

To evaluate whether HIV reservoir or TCRR architecture relates to cognitive performance, we modeled associations of global and domain deficit scores with HIV DNA/RNA levels and TCRR metrics in PBMCs and across CNS tissues.

In PBMCs, neither the size nor the activity of the HIV reservoir, nor the TCRR metrics were associated with cognitive scores. In the CNS, neither the size of the reservoir nor its transcriptional activity was associated with cognitive scores.

**Fig 4:**
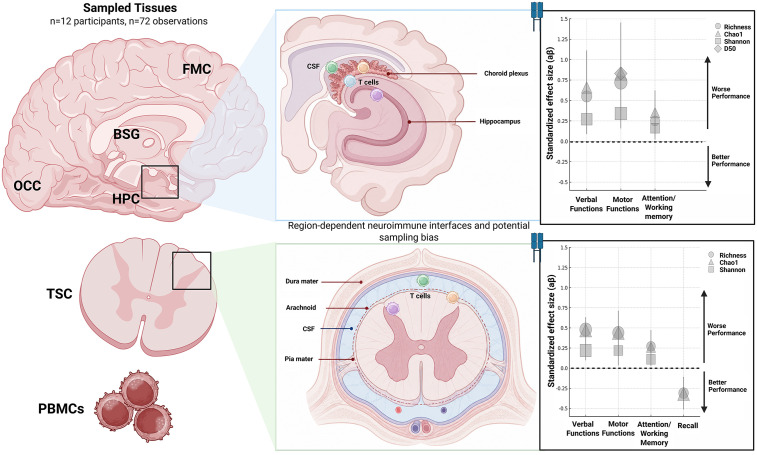
Region-resolved associations between CNS T-cell repertoire features and cognitive performance. Standardized effect sizes (aβ, with error bars representing 95% confidence intervals) from multivariable linear regression models clustered at participant level show the associations of TCRR metrics with domain deficit scores across sampled CNS regions and PBMCs. The graphs display only associations with FDR-adjusted q values <0.1. Positive aβ indicates worse cognitive performance, and negative aβ indicates better performance. All models were adjusted for sequencing depth, cause of death, last CD4 + T cell count, duration of HIV, ART regimen, and included the interaction term between TCRR metrics and CNS regions, as detailed in the methods. Middle panels illustrate region-dependent neuroimmune interfaces and bulk sampling context for the HPC and TSC. Region-targeted bulk tissue sampling may include a mixture of parenchymal (gray and white matter), perivascular, and meningeal/interface compartments, with variable contributions across regions. Although meningeal and perivascular interfaces are present throughout the CNS, their relative contribution to immune signal detection may be region-dependent, being greater in areas such as the HPC (proximity to choroid plexus) and SC (greater meningeal and CSF interface) compared with more immune-restricted regions (OCC, FMC, BSG). Accordingly, associations should not be interpreted as reflecting direct neuroanatomical localization of cognitive functions, but rather as reflecting interface-biased neuroimmune signals captured through region-specific sampling context. Abbreviations: FMC, frontal motor cortex; BSG, basal ganglia; OCC, occipital cortex; HPC, hippocampus; TSC, thoracic spinal cord; PBMCs, peripheral blood mononuclear cells; aβ, adjusted beta coefficient; TCRR, T-cell receptor repertoire. Created in BioRender. Trunfio, **M.** (2026) https://BioRender.com/m4h0p13.

In contrast, TCRR characteristics displayed domain-specific associations with cognitive scores consistently in two CNS regions only: HPC and TSC (Fig 4 and S3 Table). In both regions, higher richness, Chao1, and Shannon index were associated with worse verbal and motor scores, and Chao1 was also associated with poorer attention/working memory (with richness and Shannon showing concordant trends). Higher D50 in the HPC was also borderline correlated with worse motor scores (q = 0.065; Fig 4 and S3 Table). Conversely, higher richness and Chao1 in TSC were associated with better recall scores (Fig 4 and S3 Table). TCRR clonality showed no associations with cognitive functioning. In sensitivity analyses including the interval between cognitive assessment and autopsy as a covariate, the magnitude and direction of the observed associations were unchanged.

### 2.4. Epitope-specific TCR clonotypes show tissue- and pathogen-dependent associations with cognition

As an exploratory objective, we assessed whether the abundance of T clonotypes with known pathogen antigen specificities was associated with cognitive scores across tissues.

**Fig 5:**
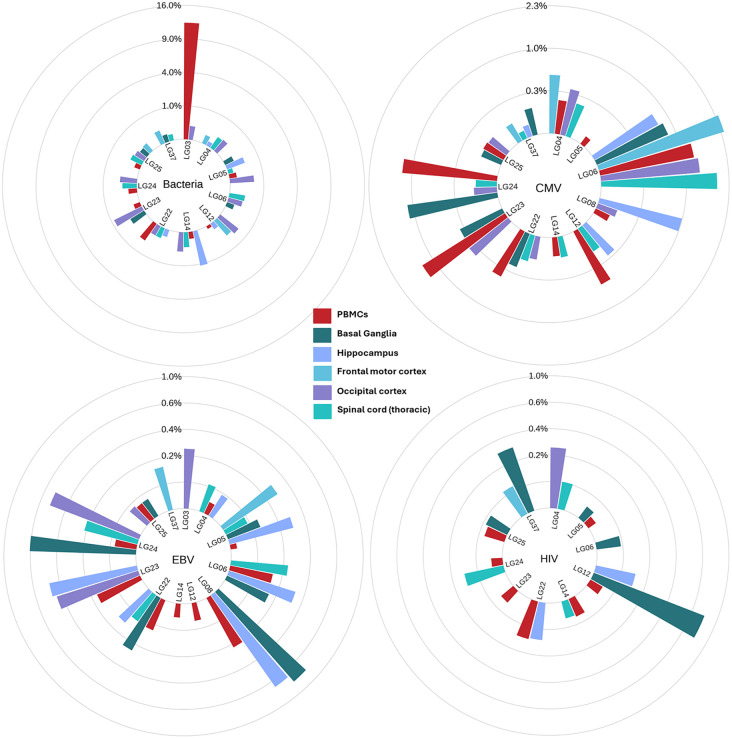
Tissue distribution of pathogen-specific T-cell clonotypes across participants. Radial diagrams display the relative abundance and anatomical distribution of epitope-specific TCR clonotypes across tissues. Each panel corresponds to one epitope category (HIV, EBV, CMV, and bacteria for 5-(2-oxopropylideneamino)-6-D-ribitylaminouracil epitope). Bars represent the relative abundance of epitope-specific clonotypes within each participant (LG#) in the corresponding tissue. Colors indicate tissues as shown in the legend. Tissues in which no epitope-specific clonotypes were detected are not displayed: 5-(2-oxopropylideneamino)-6-D-ribitylaminouracil–specific and CMV-specific clonotypes were absent in all tissues in one participant each, while HIV-specific clonotypes were absent in all tissues in two participants. Abbreviations: PBMCs, peripheral blood mononuclear cells; Bacteria, 5-(2-oxopropylideneamino)-6-D-ribitylaminouracil epitope target; TCR, T-cell receptor.

Epitope-specific clonotype distributions varied across participants and tissues, consistent with individualized antigen exposure histories (Fig 5): CMV-, EBV-, HIV-, and 5-(2-oxopropylideneamino)-6-D-ribitylaminouracil-(5-OP-RU)-specific clonotypes were detected across most tissues, with their abundance varying between participants and regions. 5-OP-RU is a riboflavin-derivative antigen from bacterial metabolism presented by the non-polymorphic MHC class I–related molecule MR1. No significant differences across CNS regions or between PBMCs and the CNS were observed in CMV-, EBV-, HIV-, or 5-OP-RU-specific clonotype representation, except for a higher number of CMV-specific T clonotypes in PBMCs compared with all CNS regions (all q < 0.02).

Significant associations between the abundance of pathogen-specific T-cell clonotypes and cognitive scores were observed in CNS tissues (Table 1). In BSG, a higher number of HIV-specific T clonotypes was associated with better GDS (aβ −0.30 p = 0.008, q = 0.040), better processing speed (aβ −0.33, p = 0.038, q = 0.190), and better attention/working memory scores (aβ −0.48, p = 0.002, q = 0.010), whereas more CMV-specific T clonotypes were associated with worse recall (aβ 0.49, p = 0.029, q = 0.145). In TSC, more CMV-specific T clonotypes were associated with worse learning scores (aβ 0.41, p = 0.015, q = 0.075), whereas in FMC, more 5-OP-RU-specific T clonotypes were associated with better motor functioning (aβ −0.85, p = 0.010, q = 0.050). In PBMCs, no associations between the number of epitope-specific T clonotypes and cognitive scores were observed. In sensitivity analyses including the interval between cognitive assessment and autopsy as a covariate, the magnitude and direction of the observed associations were unchanged.

**Table 1:** Significant associations between the number of epitope-specific TCR clonotypes and cognitive performance across CNS regions.

Tissue	Predictor	Deficit score	aβ (95%CI)	P	FDR
**BG**	CMV-specific clonotypes, n	Recall	0.49 (0.06; 0.93)	0.029	0.145
	HIV-specific clonotypes, n	Global Deficit Score Processing speed Attention/working memory	-0.30 (-0.50; -0.10) -0.33 (-0.63; -0.02) -0.48 (-0.73; -0.22)	0.008 0.038 0.002	0.040 0.190 0.010
**FMC**	5-OP-RU-specific clonotypes, n	Motor functioning	-0.85 (-1.44; -0.25)	0.010	0.050
**TSC**	CMV-specific clonotypes, n	Learning	0.41 (0.10; 0.73)	0.015	0.075

Adjusted beta coefficients (aβ, and 95% confidence intervals, 95%CI) from multivariable linear regression models examining the association between the number of epitope-specific TCR clonotypes and cognitive performance across the CNS. Models were adjusted for sequencing depth, total number of T clonotypes, CD4 ⁺ T-cell count, duration of HIV infection, and cause of death, as detailed in the Methods. Cognitive scores were already corrected for age, sex, education, race and ethnicity. Positive aβ values indicate worse cognitive performance, whereas negative values indicate better performance. Only associations reaching nominal statistical significance (p < 0.05) are shown; corresponding false-discovery rate–adjusted p-values (FDR) are reported. Abbreviations: aβ (95%CI), adjusted beta coefficients (95% confidence intervals); BG, basal ganglia; FMC, frontal motor cortex; TSC, thoracic spinal cord; 5-OP-RU, 5-(2-oxopropylideneamino)-6-D-ribitylaminouracil. Given the exploratory nature of these analyses and the limited sample size, these findings should be interpreted cautiously.

Abundance of HIV-specific T clonotypes was not associated with measured HIV reservoir metrics in models adjusted for sequencing depth, total number of T clonotypes, tissues, age, duration of infection, CD4^+^ counts, and cause of death.

## 3. Discussion

We integrated quantitative measures of HIV burden with TCRR profiling across five distinct CNS regions and peripheral blood and related these viro-immunological features to antemortem cognitive performance. In PWH on long-term suppressive ART, our findings indicate that adaptive T cell immune architecture within the CNS, rather than in periphery, and rather than CNS and peripheral HIV burden, tracks with cognitive performance. Moreover, pathogen-imprinted TCRR signatures varied within the CNS and showed tissue- and pathogen-specific associations with cognition, suggesting specificity of pathogen-immune–cognition relationships across CNS compartments.

We hypothesized that a larger and more transcriptionally active HIV reservoir in the CNS would be associated with worse cognitive performance, due to ongoing viral expression, immune activation, and neurotoxicity of viral products. However, we did not find associations between HIV metrics and cognition, despite detectable levels of HIV DNA and RNA across all regions. While neurological effects of uncontrolled viral replication within the CNS are well documented, [34–36] and *in vitro* studies demonstrate neurotoxicity of HIV transcripts and proteins even in the absence of productive replication, [37] human data linking quantitative measures of HIV persistence to cognitive impairment under suppressive ART are limited. One post-mortem study reported no association between proviral burden in frontal white matter and cognitive impairment. [12] Notably, intact proviral levels correlated with neuroinflammatory gene expression, [12] suggesting that the fraction of proviruses capable of transcriptional activity may be more closely linked to CNS injury. We likewise found no associations of cognition with total HIV DNA, and we further tested whether CNS HIV transcriptional activity or 2LTR HIV DNA, a proxy of HIV residual replication under ART, tracked with cognition and found no such associations. HIV reservoir metrics were also unrelated to the TCRR features, arguing against a model in which HIV indirectly affects cognition through its effects on TCRR. Another prior longitudinal study reported that HIV DNA levels in PBMCs were not associated with cognitive impairment; rather, within-individual changes in HIV DNA levels over time tracked with verbal and motor trajectories.[38] Compared to our participants, this prior cohort included individuals with shorter ART exposure (2 vs 18 years) and variable viral control during the follow-up, a context in which the reservoir size is more dynamic.[39] Altogether, these findings suggest that static quantitative measures of HIV persistence and activity may have limited explanatory value for cognitive outcomes under long-term viral suppression. In this context, proviral burden and snapshot measures of residual viral transcription may be insufficient to induce CNS injury detectable at the clinical level. Alternatively, total HIV DNA and bulk measures of HIV persistence may not fully capture the fraction of virus that is immunologically active or relevant for CNS injury. While we quantified usGag and msTat/Rev RNA, these bulk measurements reflect transcriptional activity at the time of death and may not adequately capture low-level, spatially restricted, or intermittent antigen expression that is most relevant to immune recognition under suppressive ART. Consistent with this, a similar pattern was observed across CNS tissues, with total HIV DNA associating with usGag but not with msTat/Rev RNA, and early and late transcriptional markers largely uncoupled, indicating that initiation of transcription may relate to reservoir size, whereas progression to later transcriptional stages is not sustained under suppressive ART. As such, total HIV DNA and RNA-based measures may not reflect the antigenic stimuli driving local T-cell responses, potentially explaining the dissociation between HIV reservoir measures and TCRR features observed here. Furthermore, comparable levels of proviral persistence and activity may be associated with markedly different degrees of immune activation across individual, as several factors (e.g., comorbidities, medications, co-infections, and genetic differences) shape their relationship.[7,40] Lastly, although both the HIV reservoir and TCRR are shaped by infection history and tissue context, TCRR architecture reflects the composition and distribution of local T cell responses and integrates cumulative antigenic exposure, which may provide a complementary and temporally integrated readout of the neuroimmune environment and longer-term immune dynamics. Future studies integrating measures of intact and inducible reservoir with temporally resolved immune profiling (e.g., transcriptomics) will be necessary to better resolve the relationship between viral persistence, HIV-specific immune responses, and CNS outcomes.

Contrary to our hypothesis, higher TCRR richness and diversity within the CNS were associated with poorer cognitive performance across multiple domains, except for higher richness in the TSC associating with better recall. The direction of this association appears to contrast with prior literature in population without HIV (e.g., Alzheimer’s disease), [17,21] where reduced diversity or oligoclonality has been associated with worse neurological features. However, these prior observations derive primarily from peripheral blood and may not translate to the CNS, where immune composition, antigen exposure, and compartmental constraints differ fundamentally. In HIV and other chronic viral infections, prior studies showed clonal expansions, repertoire skewing, and reduced breadth, [28–30,41] thereby we anticipated that lower richness and diversity would be associated with worse cognition, whereas broader repertoires would reflect better preserved immune homeostasis. However, also these studies were performed in peripheral blood.[28–30,41] In the CNS, that is normally tightly regulated and poor in T cells [42], higher TCRR richness and diversity may reflect increased permeability of neuroimmune interfaces, enhanced T-cell recruitment and retention, broader antigenic exposure, and ultimately “immune crowding” [43]. Because we could not quantify immune cell density or perform phenotypic characterization, we cannot determine whether increased richness reflects a higher number of infiltrating T cells, a broader distribution of clonotypes within a stable population, or both. Complementarily, higher TCRR richness and diversity in the CNS may reflect also “antigenic overload” due to impaired antigenic clearance, higher burden of co-infections, increased microbial translocation, [7,44] or dysregulated responses to self-antigens. All these carry detrimental consequences for neuronal and glial function, and have been previously linked to cognitive impairment.[8,7,45,46] This interpretation is also consistent with other evidence in PWH that found higher TCRR diversity in the blood to be associated with increased expression of T cell activation and IFN-γ–associated genes, [30] suggesting a link with heightened immune activation. Thus, in ART-suppressed PWH, higher CNS TCR richness and diversity may reflect neuroimmune activity associated with poorer cognitive outcomes. Because we lack normative reference ranges for TCRR metrics and comparison groups of people without HIV and PWH off ART, we cannot determine whether the observed values reflect normal variation or a fundamentally altered state. Nevertheless, the observed associations indicate that variation within the range observed in PWH on suppressive ART remains biologically meaningful and tracks inter-individual cognitive differences. Taken together, these findings suggest that the TCRR-cognition signal may be compartment-specific, and that hypotheses derived from peripheral blood may not directly apply to the CNS.

The CNS TCRR was not only distinct from peripheral blood, but also compartmentalized across CNS regions, despite broadly similar aggregate richness, diversity, and clonality. Low-to-moderate overlap in clonotype frequency distributions between CNS region pairs suggests that each anatomical site harbors partially distinct T cell populations and antigenic experiences, consistent with prior work demonstrating TCRR compartmentalization between blood and peripheral tissues.[47,48] Our data describe this regional immune compartmentalization also in the CNS of PWH.

Associations between TCRR and cognition emerged preferentially in specific regions such as the HPC and TSC. This selective signal may reflect differential sampling of tissue immunological niches. The HPC is a region where immune-mediated perturbation have been extensively characterized, given its high synaptic plasticity, dense microglial network, and susceptibility to inflammatory cytokines.[49,50] Experimental work has shown that T cell–derived signals can bidirectionally modulate hippocampal neurogenesis, synaptic remodeling, and cognitive functions, depending on the balance between regulatory and inflammatory pathways.[22,27,51,52] Moreover, HPC lies in close proximity to choroid plexus, a site of active immune surveillance enriched in T cells.[42,53] As a result, bulk hippocampal sampling may partially capture immune populations associated with border compartments, where T cell activity is richer and closely coupled to dynamic cognitive circuits, rather than exclusively reflecting parenchymal processes intrinsic to HPC function. In fact, these findings should not be interpreted as reflecting anatomical localization of cognitive functions to these regions. Similarly, compared with brain, TSC exhibits greater immune accessibility, higher relative contribution of meningeal and perivascular immune compartments, and closer integration with CSF circulation [54–57]. These features could potentially weight tissue sampling and bulk tissue TCRR profiling toward border-associated T-cell populations, where immune traffic and antigenic sampling are more pronounced. In contrast, cortical regions and BSG are characterized by tighter immune exclusion and lower T cell density, [42,53] potentially being less permissive to detectable TCRR–cognition coupling in bulk tissue analyses. Taken together, we interpret the apparent hippocampal and spinal specificity of the TCRR-cognition relationship as a reflection of T cells sampled at periphery-CNS interfaces, in line with prior evidence of T cells modulating neuroinflammation and cognitive functions from the leptomeninges and choroid plexus.[58–61] Overall, these associations should be interpreted as signatures of region-specific immune accessibility and interface dynamics, rather than as evidence of direct neuroanatomical mapping of cognitive functions. This framework explains why TCRR features measured in the HPC and TSC associated with cognitive functions that do not have a direct neuroanatomical substrate in these regions, and why higher TCRR richness in the TSC was associated with better recall, in contrast to its association with poorer performance in other domains. This divergence could be interpreted as the net effect of heterogeneous coexisting T-cell subpopulations captured by bulk TCRR profiling across anatomically and functionally distinct compartments (e.g., meningeal, perivascular, parenchymal), rather than as bidirectional effects of a single biological entity. The mechanistic interpretations proposed here, including increased immune cell trafficking, antigenic exposure, and compartment-specific immune activation, should be considered hypothesis-generating and not evidence of causality, as they cannot be directly validated in the absence of complementary cellular and molecular measurements. Future studies using spatial immunophenotyping, single-cell or single-nucleus transcriptomic profiling, complemented by vascular or barrier markers across regions, are warranted to verify such hypothesis, localize, and functionally characterize border-associated versus parenchymal T cells.

Beyond global TCRR architecture, our epitope-inference analyses suggest that the antigenic imprint of T cells in CNS tissues is also functionally relevant to cognition, with distinct pathogen-specific T responses exerting divergent effects. These associations emerged exclusively in CNS tissues, suggesting that local antigen-experienced T-cell populations, rather than peripheral responses, are more closely linked to cognitive performance. Specifically, in the BSG, a greater abundance of HIV Gag-specific T-cell clonotypes was associated with better global cognition, processing speed, and attention/working memory, independently of HIV reservoir. This dissociation suggests that the presence of HIV-specific clonotypes may reflect immune surveillance or containment mechanisms that are not directly captured by bulk measures of viral burden, which include both intact and defective proviruses. Even transcriptional metrics may not capture the fraction of virus that is antigenically relevant, as low-level or intermittent expression to sustain T-cell responses may not be detected in cross-sectional measurements. These T cells may represent effective immune surveillance or containment, potentially limiting inflammation, HIV activity, and local injury, consistent with prior evidence of effective HIV control by Gag-specific CD4+ and CD8 + T cells [62–64]. The dissociation between these clonotypes and HIV reservoir further supports a shift away from a reservoir-centric model of HIV-associated neuro-injury toward models emphasizing immune composition, antigenic history, co-infections, and surveillance dynamics under long-term viral suppression. As noted above, these associations should not be interpreted as reflecting direct functional roles of the sampled anatomical regions in the corresponding cognitive domains. We interpret them as region-dependent immune signatures shaped by differences in immune accessibility, antigen exposure, and T-cell trafficking across CNS compartments, collectively reflecting system-level neuroimmune processes. The abundance of epitope-specific clonotypes in each region may reflect where these cells are detectable, and potentially preferentially retained, at the time of sampling, rather than the site where their functional effects on cognition necessarily occur. For example, the association between higher abundance of HIV-specific clonotypes in the BSG and better processing speed and attention/working memory is unlikely to indicate a region-specific functional effect. Instead, it may reflect more effective anti-HIV immune surveillance at the CNS level, with HIV-specific T cells more easily detectable, accumulating or persisting in subcortical regions, that have historically been implicated as preferential sites with significant antigenic exposure in untreated infection and vulnerability to HIV-related injury.[65,66] Within this framework, the BSG may represent a site of enhanced detectability of these clonotypes due to local antigenic cues, whereas their relationship with neurocognitive outcomes may reflect distributed or system-level neuroimmune dynamics.

CMV-specific T clonotypes were associated with worse recall in BSG and worse learning performance in TSC. This finding aligns with a large literature implicating CMV as a major driver of immune aging, clonal expansion, chronic inflammation, and comorbidities including cognitive impairment.[7,67] CMV-specific T cells often exhibit senescent or cytotoxic phenotypes and can secrete pro-inflammatory mediators even in the absence of overt viral reactivation.[68] In people with and without HIV, CMV seropositivity and expanded CMV-specific T cell responses have been variably linked to cognitive decline, immune senescence, and neuroinflammation.[69–71] Together, these observations suggest that antigen-experienced T cells may differ in their neurobiological associations depending on pathogen specificity and immune context: HIV-specific clonotypes under longstanding suppressive ART may reflect a more regulated, antigen-focused immune control, whereas expanded CMV-specific clonotypes may index recurrent or prolonged CMV reactivations, immune exhaustion, and sustained inflammation. Future studies with improved epitope resolution are required to determine whether responses targeting distinct epitopes within the same pathogen exhibit different neurobiological associations. Lastly, we observed an association between higher abundance of MAIT-like (5-OP-RU–specific) clonotypes in the FMC and better motor performance, suggesting a potential neuroprotective role for these innate-like T cells. MAIT cells have been shown to exert a context-dependent role in CNS disorders, with evidence for both neuroprotective and neuro-inflammatory activity.[72] In HIV, MAIT cells undergo profound quantitative depletion and functional reprogramming, with unclear long-term consequences.[73,74] Our findings are consistent with experimental evidence that MAIT cells in the CNS support meningeal barrier integrity, suppress oxidative injury, and preserve cognitive performance.[75] Because MAIT-like clonotypes were inferred based on TCR specificity for 5-OP-RU rather than direct phenotypic characterization, these findings should be interpreted as reflecting MAIT-like activity rather than definitive MAIT cell identity. Altogether, these pathogen-dependent associations are compatible with emerging keystone epitope concepts, in which persistent infections can entrain stable, compartmentalized antigen-specific T-cell hierarchies that shape local immune tone and clinical phenotypes independently of bulk pathogen burden [76,77].

Among the strengths of the study is the unique rapid-autopsy cohort with paired detailed antemortem cognitive characterization and post-mortem sampling of multiple anatomically distinct CNS regions, a design that is not feasible in living cohorts and rarely achievable even in post-mortem studies. Second, we integrated quantitative HIV reservoir measurements, TCRR sequencing, and epitope-specific TCRR inference within the same tissues, enabling a comprehensive, immune-genomic view of CNS T-cell architecture. Third, the use of region-resolved statistical models with participant-level clustering allowed us to leverage within-individual anatomical variation while controlling for key confounders, increasing inferential robustness despite the limited sample size. Although substantial inter-host variability, the depth of multi-regional sampling partially mitigated this by enabling within-individual comparisons. Lastly, the persistence of significance after FDR correction supports strong biological signal.

Among the limitations, the sample size was small, reflecting the rarity of CNS tissue linked to viro-immunological and cognitive assessments. This limits statistical power and although participant-clustered robust standard errors were used to account for repeated measurements, the limited number of clusters may reduce the precision of variance estimates. However, the multi-regional sampling partially compensated through the higher number of observations, and the primary conclusions rely on consistent patterns rather than isolated associations; furthermore, sensitivity analyses excluding the participant with amyotrophic lateral sclerosis did not alter results. A second important limitation is the absence of soluble or transcriptomic inflammatory measurements. As a result, we cannot directly link TCRR features to specific inflammatory pathways or quantify the degree of neuroinflammation within each tissue. However, inflammatory markers are highly dynamic and may be influenced by perimortem factors, including terminal illness and cause of death, whereas cognitive assessments were performed months prior to death and appeared on average stable over at least the last 2 years of life. In this context, TCRR architecture may be more temporally aligned with cognition than highly dynamic inflammatory markers and it may provide a more integrative readout of neuroimmune activity, but mechanistic interpretation remains indirect and without defined immune mediators and pathways. Future studies combining TCRR profiling with cytokine measurements, spatial transcriptomics, and single-cell approaches will be necessary to resolve these relationships. Third, CNS regions were analyzed as bulk tissue without separation of gray and white matter compartments, and deep white matter was not systematically sampled as a distinct anatomical region. With the exception of TSC, all sites showed gray matter predominance. HIV-associated neurocognitive disorders have been linked to pathology in deep white matter and selected subcortical regions [78]. As such, our study cannot directly address white matter–predominant mechanisms of injury and precludes resolution of tissue-specific differences arising from distinct T-cell subpopulations and their local microenvironmental niches [79]. Instead, our findings should be interpreted as reflecting immune architecture within bulk region-targeted tissue, encompassing gray matter and immediately adjacent white matter, and does not allow distinction between cortical, juxtacortical, and deep white matter T cells. For the same reason, we could not distinguish the relative contribution of CD4+ and CD8 + T-cell populations to the observed repertoire features. Peripheral CD8 + T-cell counts and CD4/CD8 ratios were available only in a subset of participants, precluding their inclusion in the models without substantial loss of power. As such, the subset-specific drivers of the observed TCRR associations, including the relative contribution of cytotoxic versus regulatory immune components, cannot be determined. Fourth, the cross-sectional design precludes determination of the temporal sequence between TCRR and cognition: we cannot establish whether the observed TCRR features preceded cognitive impairment, contributed to its development, or instead reflect persistent immune surveillance or adaptation following prior CNS injury. Thus, some of the observed associations may represent immune responses to historical or cumulative damage rather than drivers of ongoing pathology, or both. This limitation is partially mitigated by the nature of TCRR itself: it reflects cumulative antigenic exposure and long-term immune dynamics. Longitudinal studies in peripheral blood indicate that TCRR architecture evolves gradually over years to decades, largely driven by aging and chronic antigen exposure [80]. Over shorter timeframes (months to one year), dominant clonotypes and overall repertoire structure appear relatively stable [81], despite ongoing antigen-driven fluctuations or HIV infection [30,82]. Unlike soluble inflammatory markers, which can shift over hours to days, the clonal composition of T cell populations, especially in an immunologically privileged compartment like the CNS, reflects cumulative antigen exposure and clonal selection over months to years. Significant reshaping of the CNS TCR repertoire would require sustained antigenic drive or major immune reconstitution events, neither of which is expected in ART-suppressed individuals over short timeframes; however, longitudinal data in CNS tissues are lacking. In this context, although TCRR was measured at a single time point, its short-term stability in peripheral compartments, together with the limited change in cognitive status in our cohort, in line with prior longitudinal studies showing relative stability of cognitive trajectories over few years in PWH [83,84], support the plausibility of biologically meaningful associations within this timeframe, although causal relationships cannot be inferred. Sixth, epitope-specific TCRR inference relies on reference databases with uneven microbial coverage. While we restricted analyses to well-represented epitopes, misclassification and under-representation are possible. Thus, the epitope-specific findings should be viewed as biologically plausible signals rather than exhaustive antigen mapping. Finally, older white men, all with terminal illness, predominantly comprised the cohort, limiting generalizability.

In conclusion, this study provides human tissue–based evidence that CNS-localized T-cell immune architecture and its antigenic imprint are associated with cognitive variability independently of quantitative measures of HIV persistence and transcriptional activity in virally suppressed PWH. While HIV persistence in the CNS remains a central challenge for eradication efforts, our findings support a conceptual shift towards a framework in which the architecture, composition, and specialization of local T-cell populations represent a critical and previously underexplored dimension of brain health beyond HIV alone.

## 4. Methods

### 4.1. Ethics statement

The Last Gift and CNTN studies were approved by the University of California San Diego Human Research Protections Program (IRB#s 160563, 171024). All participants provided written informed consent for antemortem data collection and post-mortem rapid research autopsy. All procedures adhere to the Declaration of Helsinki.

### 4.2. Study design and participants

We performed a cross-sectional, multi-compartmental immune-genomic study nested within the Last Gift study, an end-of-life research program at UCSD, designed to characterize HIV persistence across tissues in PWH who altruistically donate their bodies for rapid post-mortem tissue recovery. [33]

Participants contributed antemortem clinical evaluations, cognitive assessments, and blood samples (PBMCs), along with post-mortem tissue specimens from five CNS regions (HPC; FMC; BSG; OCC; TSC) collected within six hours of death. CNS samples were obtained as region-targeted bulk tissue, without separation of gray and white matter compartments. As such, samples included grey matter and variably adjacent white matter, but deep white matter was not systematically or independently sampled as a distinct anatomical compartment. By protocol, all brain tissue samples were obtained from the right hemisphere only, to ensure consistency. Consequently, this study was designed to assess regional immune-genomic signatures in bulk CNS tissue, rather than tissue compartment-specific neuropathology and specific T-cell populations contributing to TCRR features.

Eligible participants were ≥18 years old, had confirmed HIV, maintained plasma HIV RNA < 50 copies/mL for ≥6 months before cognitive testing, and underwent a cognitive evaluation within one year before death. Participants had no major CNS confounding conditions (e.g., CNS malignancy, infections, untreated psychiatric disorders), except for one individual with amyotrophic lateral sclerosis. Given the rarity of rapid-autopsy datasets, and to preserve statistical power, this participant was retained; however, all analyses were repeated excluding this individual, with no meaningful change in effect direction, magnitude, or significance (n = 11). We therefore present results for the full cohort.

### 4.3. Medical and neurocognitive assessment

Participants were comprehensively assessed, including demographic characteristics, medical history, and physical examination at multiple time points before death (PBMCs used in this study were obtained from the last blood collection before death). Through co-enrollment in the CNTN, a clinical site of the National NeuroHIV Tissue Consortium, all participants completed a neurocognitive assessment, which evaluated seven cognitive domains: Verbal fluency (Controlled Oral Word Association Test; Animal Fluency), Executive functioning (Trails B test; Wisconsin Card Sorting Test-64, Perseverative Responses; Stroop Color and Word Test, Color-Word Trial), Processing speed (Trails A test; WAIS-III Digit Symbol; WAIS-III Symbol Search; Stroop Color Trial), Learning (Hopkins Verbal Learning Test-Revised [HVLT-R]-Total Learning; Brief Visual Memory Test-Revised [BVMT-R]-Total Learning), Recall (HVLT-R-Delayed Recall; BVMT-R-Delayed Recall), Attention/Working Memory (WAIS-III Letter Number Sequencing; Paced Auditory Serial Addition Test-50), and Motor functioning (Grooved Pegboard Dominant and Non-dominant Hand). Raw test scores were transformed into normally distributed T-scores, which are demographically adjusted for age, education, sex, and ethnicity/race using established normative data from the general population.[85–87] T-scores were transformed into Domain deficit scores and the GDS as previously described, [88] with scores >0.5 and ≥0.5 indicating domain and global impairment, respectively.[88] The interval between the neurocognitive assessment and death was calculated for each participant. For participants with more than one neurocognitive assessment available, we also calculated change in global and domain deficit scores between the final and the preceding assessment, normalized by the interval between assessments, for descriptive purposes of the cognitive trajectory.

### 4.4. HIV reservoir characterization

Methods for HIV reservoir characterization within the Last Gift study have been extensively described in prior publications.[33,89] Briefly, snap-frozen tissues were mechanically homogenized and processed using the QIAamp DNA Mini Kit (DNA) and RNeasy Mini Kit (RNA) (Qiagen). Total DNA and RNA concentrations were determined using NanoDrop One (ThermoFisher Scientific), and RNA integrity was verified using a TapeStation (Agilent Technologies). For HIV DNA, ddPCR reactions targeting 2-long terminal repeat (2LTR) and Gag region (skGag) were run in triplicate using multiplexed Gag_HEX and 2LTR_FAM primer/probe sets on the QX200 system (Bio-Rad). HIV DNA copy numbers were normalized to one million cells based on RPP30. For HIV RNA, samples underwent DNase treatment followed by reverse transcription, and cDNA was generated using validated protocols. UsGag and msTat/Rev transcripts were quantified by ddPCR (Gag_HEX and TatRev_FAM primer/probe sets) in triplicate. Values below the detection limit were considered biologically zero.

### 4.5. TCRR characterization

Bulk high-throughput sequencing of TCRR was performed on bulk CNS tissues and matched PBMCs using the Archer Immunoverse-HS TCR reagents for Illumina (P/N dSK0159; NovaSeq 6000) on the IMMUNOVerse platform (Vanderbilt University Medical Center). Because tissues were not sorted into T-cell subsets, TCRR profiles reflect the combined contribution of CD4+ and CD8 + T cells. Library preparation followed the Archer Immunoverse-HS TCR protocol for Illumina and used Anchored Multiplex PCR with panel-specific unidirectional gene-specific primeres, molecular barcode adapters, and nested PCR to generate target-enriched libraries for Illumina sequencing. Resulting FASTQ files underwent standardized quality control and annotation using MiXCR [90] and analyzed with the Immunarch R package (v0.10.3) [91] for CDR3 extraction, clonotype assembly, identification of unique productive rearrangements, and computation of TCRR metrics. Analyses were restricted to in-frame productive rearrangements. For each sample, the TCRR was described through:

Richness: richness, as the total number of unique productive T cell receptor clonotypes; Chao1, as a bias-corrected richness metric accounting for rare clonotypes

Diversity**:** Shannon entropy, as the number of unique clonotypes and their relative frequencies, reflecting the balance between dominant and rare clones.

Clonality: a measure of repertoire skewing toward expanded clonotypes, with higher values indicating dominance of a limited number of highly expanded clones and lower values reflecting a more even distribution across clonotypes; D50 index, as the fraction of the most abundant clonotypes required to account for 50% of all productive CDR3 reads, with lower values indicating a repertoire dominated by few expanded clones.

Inter-tissue TCRR similarity: Morisita–Horn index**,** to quantify the overlap in clonal frequency distributions between pairs of tissues. The index ranges from 0 (no shared composition) to 1 (identical frequency distributions).

### 4.6. Inference of TCRR antigen specificity

Antigen specificity of TCRRαβ clonotypes was inferred using ImmuneWatch DETECT (v1.0; ImmuneWatch BV, 2024), which maps CDR3 amino-acid sequences and V(D)J gene usage to experimentally validated TCRR–epitope pairs curated from immune repertoire datasets.[92] Because the reference database is largely derived from studies on SARS-CoV-2, coverage for other pathogens is uneven, limiting the selection of pathogens and related epitopes of interest. Specifically, among viruses, EBV, CMV, VZV, HCV, HBV, and HIV had epitope coverage. Validated epitope-level assignments were aggregated into pathogen-level categories: a) HIV-specific (Gag, p24); b) CMV-specific (Phosphoprotein 65, Phosphoprotein 50, Trans-activating transcriptional regulatory protein IE1); c) EBV-specific (EBNA3A, LMP2A, BMLF1, BZLF1, BRLF1). Only epitopes matching with sufficient pooled representation were retained (≥5% of samples; HCV, HBV, and VZV were not retained).

Host-derived epitopes were considered in relation to CNS biology (e.g., CD1d:sulfatide of human antigen myelin-glycosphingolipid) but ultimately excluded due to low representation. Lastly, T clonotypes specific for 5-OP-RU, a riboflavin-derivative antigen from bacterial metabolism presented by the non-polymorphic MHC class I–related molecule MR1, were considered, and included. Given that 5-OP-RU is the MR1-presented antigenic ligand that uniquely activates mucosal-associated invariant T (MAIT) cells, [93,94] clonotypes assigned to this epitope were interpreted as MAIT-like cells.

For each sample, we quantified the absolute number of clonotypes specific to each epitope category by summing all clonotypes that met the category-specific criteria. Total number of clonotypes per sample was used to adjust the models that had epitope-specific clonotypes as predictor.

### 4.7. Statistical analysis

Continuous and categorical variables are reported as mean±standard deviation, median (interquartile range), and number (percentage), as appropriate. Continuous variables were transformed to reduce skewness in distribution. For reservoir metrics, all values were log10-transformed after adding a constant offset (+0.1 for HIV DNA and +0.01 for RNA) to accommodate values below the limit of detection. Available measurements across study samples are reported in S4 Table. Participants with missing values were excluded only from the corresponding models: PBMC models for HIV DNA or RNA included 11 observations each (out of 12); CNS models for HIV DNA and those for HIV RNA included 60 and 59 observations (out of 60), respectively; CNS models for TCRR included 58 observations (out of 60).

Associations between continuous domain and global deficit scores and predictors were evaluated using multivariable linear regression models with participant-clustered robust standard errors to account for repeated measures within individuals. Models for PBMCs included a single observation per participant, and therefore participant clustering was excluded from the model. Models testing interactions between the predictors and tissues were followed by estimation of marginal effects and pairwise contrasts of margins. For models assessing the association between domain and global deficit scores and TCRR or HIV reservoir metrics, covariates included sequencing depth, last CD4 + count, duration of HIV, cause of death, and the interaction term with tissue type. For models evaluating domain and global deficit scores in relation to the number of epitope-specific clonotypes, covariates included total number of clonotypes, sequencing depth, last CD4 + count, duration of HIV, cause of death, and the interaction term with tissue type. The same approach was used to compare TCRR metrics, reservoir metrics, and number of epitope-specific clonotypes across tissues instead of paired tests, to better account for confounding factors (age, sex, sequencing depth, total number of productive clonotypes, CD4 + count, duration of HIV infection, cause of death). CD8 + T-cell counts and CD4/CD8 ratios were summarized descriptively but were not included in primary multivariable models because they were available only in a subset of participants, which would have further reduced model stability.

Sensitivity analyses additionally adjusted for the interval between final cognitive assessment and death to evaluate whether temporal separation between cognitive testing and post-mortem tissue sampling influenced the observed associations. To account for multiple comparisons, p values were adjusted using the Benjamini–Hochberg false discovery rate procedure. False discovery rate–adjusted p values (q values) are reported throughout.

All analyses were conducted using R software (v4.4.1, R Core Team, 2025) and Stata v19 (StataCorp LLC., College Station, TX, US).

## Supporting information

S1 Table Descriptive statistics for HIV and TCR metrics across tissues. (DOCX)

S2 Table Significant correlations among HIV reservoir measures across central nervous system tissues. (DOCX)

S3 Table Significant associations between TCR metrics and cognitive domains across tissues. (DOCX)

S4 Table Available measurements of HIV and TCR across the study samples. (DOCX)

S1 Fig Graphical abstract. Conceptual overview of the study highlighting representative significant associations between T-cell receptor repertoire features, target epitopes, and cognitive outcomes across central nervous system tissues in people with HIV.
(TIF)

S1 Data Clinical, cognitive, TCRR, and HIV reservoir data supporting the findings of the manuscript. (XLSX)
