# Supplementary material for: Central Nervous System T-cell immune architecture, and not HIV burden, tracks with cognition under long-term viral suppression
Source: PLoS Pathog. 2026 Jun 15;22(6):e1014351. doi: 10.1371/journal.ppat.1014351 (PMC13286276; doi:10.1371/journal.ppat.1014351)
Supplement: S1 Table — (DOCX) [file ppat.1014351.s001.docx]

**S1 Table. Descriptive statistics for HIV and TCR metrics across tissues**

| **Site** | **Stat.** | **Richness** | **Shannon** | **Chao1** | **D50** | **Clonality** | **HIV DNA** | **2LTR** | **usGag** | **msTat/Rev** |
| --- | --- | --- | --- | --- | --- | --- | --- | --- | --- | --- |
| **PBMCs** | **Median** | 15,129 | 6.6 | 15,524.6 | 52 | 0.206 | 52.6 | 0.01 | 0.08 | 0.04 |
|  | **I** | 631 | 5.4 | 921.5 | 35 | 0.160 | 13.9 | 0 | 0.018 | 0.02 |
|  | **III** | 38,276 | 8.1 | 40,146.8 | 676 | 0.291 | 128.7 | 5.85 | 0.30 | 0.10 |
| **BSG** | **Median** | 283.5 | 4.6 | 291.3 | 23 | 0.179 | 3.6 | 1.51 | 0.018 | 0.018 |
|  | **I** | 170.5 | 3.1 | 193.4 | 4.5 | 0.111 | 1.2 | 0 | 0 | 0 |
|  | **III** | 449.5 | 4.9 | 497.9 | 34.5 | 0.334 | 23.8 | 3.30 | 0.07 | 0.080 |
| **FMC** | **Median** | 217 | 4.2 | 225.3 | 18 | 0.082 | 3.6 | 0.95 | 0.017 | 0.018 |
|  | **I** | 70 | 3.9 | 109.9 | 8 | 0.061 | 0 | 0 | 0 | 0 |
|  | **III** | 353 | 5.1 | 382.2 | 41 | 0.116 | 8.4 | 5.19 | 0.019 | 0.060 |
| **HPC** | **Median** | 337 | 4.7 | 445.7 | 28 | 0.147 | 3.9 | 4.11 | 0.017 | 0.018 |
|  | **I** | 153 | 4.3 | 167.2 | 17 | 0.099 | 1.4 | 0 | 0 | 0 |
|  | **III** | 499 | 5.4 | 602.5 | 42 | 0.215 | 5.9 | 5.52 | 0.04 | 0.14 |
| **OCC** | **Median** | 333 | 4.9 | 387.6 | 32.5 | 0.137 | 3.1 | 2.91 | 0 | 0 |
|  | **I** | 199.5 | 4.6 | 213.0 | 22 | 0.078 | 1.8 | 0 | 0 | 0 |
|  | **III** | 534 | 5.1 | 615.9 | 48 | 0.153 | 7.7 | 6.91 | 0.009 | 0.045 |
| **TSC** | **Median** | 565 | 4.7 | 679.4 | 20 | 0.207 | 21.6 | 2.74 | 0.017 | 0 |
|  | **I** | 221 | 4.2 | 263.9 | 10 | 0.148 | 4.3 | 0 | 0 | 0 |
|  | **III** | 1209 | 5.3 | 1217.5 | 30 | 0.276 | 39.9 | 3.76 | 0.018 | 0.018 |

Abbreviations: I, first quartile; III, third quartile; FMC, frontal motor cortex; BSG, basal ganglia; OCC, occipital cortex; HPC, hippocampus; TSC, thoracic spinal cord; PBMCs, peripheral blood mononuclear cells.
