# Supplementary material for: Central Nervous System T-cell immune architecture, and not HIV burden, tracks with cognition under long-term viral suppression
Source: PLoS Pathog. 2026 Jun 15;22(6):e1014351. doi: 10.1371/journal.ppat.1014351 (PMC13286276; doi:10.1371/journal.ppat.1014351)
Supplement: S2 Table — (DOCX) [file ppat.1014351.s002.docx]

**S2 Table. Significant correlations among HIV reservoir measures across central nervous system tissues**

| **FMC** | **BSG** | **HPC** | **OCC** | **TSC** |
| --- | --- | --- | --- | --- |
| HIV DNA – usGag RNA  ρ 0.57, p=0.053 | HIV DNA – usGag RNA  ρ 0.64 p=0.024 | HIV DNA – usGag RNA  ρ 0.70, p=0.011  usGag RNA – msTat/Rev RNA  ρ 0.72, p=0.0077 | HIV DNA – usGag RNA  ρ 0.65, p=0.021 | HIV DNA – usGag RNA  ρ 0.55, p=0.079 |

Spearman correlation coefficients (ρ) and corresponding p-values are shown for significant (p<0.01) pairwise associations between total HIV DNA, 2- long terminal repeat (2-LTR) circular HIV DNA, unspliced HIV Gag RNA (usGag), and multiply spliced HIV Tat/Rev RNA (msTat/Rev) within each tissue. Correlations were computed separately for each anatomical site. Across all CNS tissues, total HIV DNA showed a positive association with usGag RNA, whereas no correlations were observed between HIV DNA and msTat/Rev RNA, usGag and msTat/Rev RNA (except in HPC), and all the three metrics and 2-LTR DNA. No significant correlations were observed in PBMCs. Abbreviations: FMC, frontal motor cortex; BSG, basal ganglia; HPC, hippocampus; OCC, occipital cortex; TSC, thoracic spinal cord; PBMCs, peripheral blood mononuclear cells.
