# Supplementary material for: Central Nervous System T-cell immune architecture, and not HIV burden, tracks with cognition under long-term viral suppression
Source: PLoS Pathog. 2026 Jun 15;22(6):e1014351. doi: 10.1371/journal.ppat.1014351 (PMC13286276; doi:10.1371/journal.ppat.1014351)
Supplement: S3 Table — (DOCX) [file ppat.1014351.s003.docx]

**S3 Table. Significant associations between TCR metrics and cognitive domains across tissues**

| **CNS regions** | **TCR**  **predictor** | **Cognitive outcome** | **aβ (95%CI)** | **p** | **FDR-q** |
| --- | --- | --- | --- | --- | --- |
| **Hippocampus** | **Richness**  **Chao1**  **Shannon** | **Verbal fluency** | 0.55 (0.16; 0.94)  0.66 (0.21; 1.11)  0.27 (0.091; 0.45) | 0.010  0.008  0.007 | **0.025**  **0.020**  **0.018** |
|  | **Richness**  **Chao1**  **Shannon**  **D50** | **Motor functioning** | 0.72 (0.37; 1.07)  0.83 (0.37; 1.29)  0.34 (0.16; 0.52)  0.83 (0.21; 1.45) | 0.001  0.002  0.002  0.013 | **0.005**  **0.005**  **0.010**  0.065 |
|  | **Richness**  **Chao1**  **Shannon** | **Attention/Working memory** | 0.25 (0.047; 0.45)  0.35 (0.076; 0.62)  0.16 (0.024; 0.29) | 0.020  0.017  0.025 | 0.055  **0.042**  0.062 |
| **Thoracic spinal cord** | **Richness**  **Chao1**  **Shannon** | **Verbal fluency** | 0.48 (0.33; 0.62)  0.47 (0.30; 0.63)  0.22 (0.099; 0.35) | <0.001  <0.001  0.002 | **0.005**  **0.005**  **0.010** |
|  | **Richness**  **Chao1**  **Shannon** | **Motor functioning** | 0.44 (0.18; 0.71)  0.44 (0.21; 0.66)  0.22 (0.037; 0.40) | 0.003  0.001  0.022 | **0.008**  **0.005**  0.055 |
|  | **Richness**  **Chao1**  **Shannon** | **Attention/Working memory** | 0.27 (0.076; 0.46)  0.26 (0.062; 0.47)  0.11 (0.029; 0.18) | 0.011  0.015  0.012 | 0.055  **0.042**  0.060 |
|  | **Richness**  **Chao1** | **Recall** | -0.31 (-0.51; -0.11)  -0.32 (-0.46; -0.17) | 0.006  0.001 | **0.030**  **0.005** |

Adjusted beta coefficients (aβ) from multivariable linear regression models examining the association between TCR characteristics and cognitive performance across the CNS. Models were adjusted for sequencing depth, CD4⁺ T-cell count, duration of HIV infection, cause of death, ART regimen, and included the interaction term between TCR metrics and CNS regions, as detailed in the Methods. Cognitive scores were already corrected for age, sex, education, race and ethnicity. Positive aβ values indicate worse cognitive performance, whereas negative values indicate better performance. Only associations reaching nominal statistical significance (p<0.05) are shown; corresponding false-discovery rate–adjusted q-values (FDR-q) are reported. Abbreviations: aβ (95%CI), adjusted beta coefficient (95% confidence interval).
