# Supplementary material for: Central Nervous System T-cell immune architecture, and not HIV burden, tracks with cognition under long-term viral suppression
Source: PLoS Pathog. 2026 Jun 15;22(6):e1014351. doi: 10.1371/journal.ppat.1014351 (PMC13286276; doi:10.1371/journal.ppat.1014351)
Supplement: S4 Table — (DOCX) [file ppat.1014351.s004.docx]

**S4 Table. Available measurements of HIV and TCR across the study samples**

|  | **Total HIV DNA and**  **HIV 2LTR** | **HIV _us_Gag RNA and**  **HIV _ms_Tat/Rev RNA** | **TCR sequencing** |
| --- | --- | --- | --- |
| **Basal ganglia** | 12/12 | 12/12 | 12/12 |
| **Frontal motor cortex** | 12/12 | 12/12 | 12/12 |
| **Hippocampus** | 12/12 | 12/12 | 11/12 |
| **Occipital cortex** | 12/12 | 12/12 | 12/12 |
| **Thoracic spinal cord** | 12/12 | 11/12 | 11/12 |
| **Total observations for CNS model** | 60/60 | 59/60 | 58/60 |
| **PBMCs = total observations for blood models** | 11/12 | 11/12 | 12/12 |
