# Supplementary figures and images for: Central Nervous System T-cell immune architecture, and not HIV burden, tracks with cognition under long-term viral suppression
Source: PLoS Pathog. 2026 Jun 15;22(6):e1014351. doi: 10.1371/journal.ppat.1014351 (PMC13286276; doi:10.1371/journal.ppat.1014351)

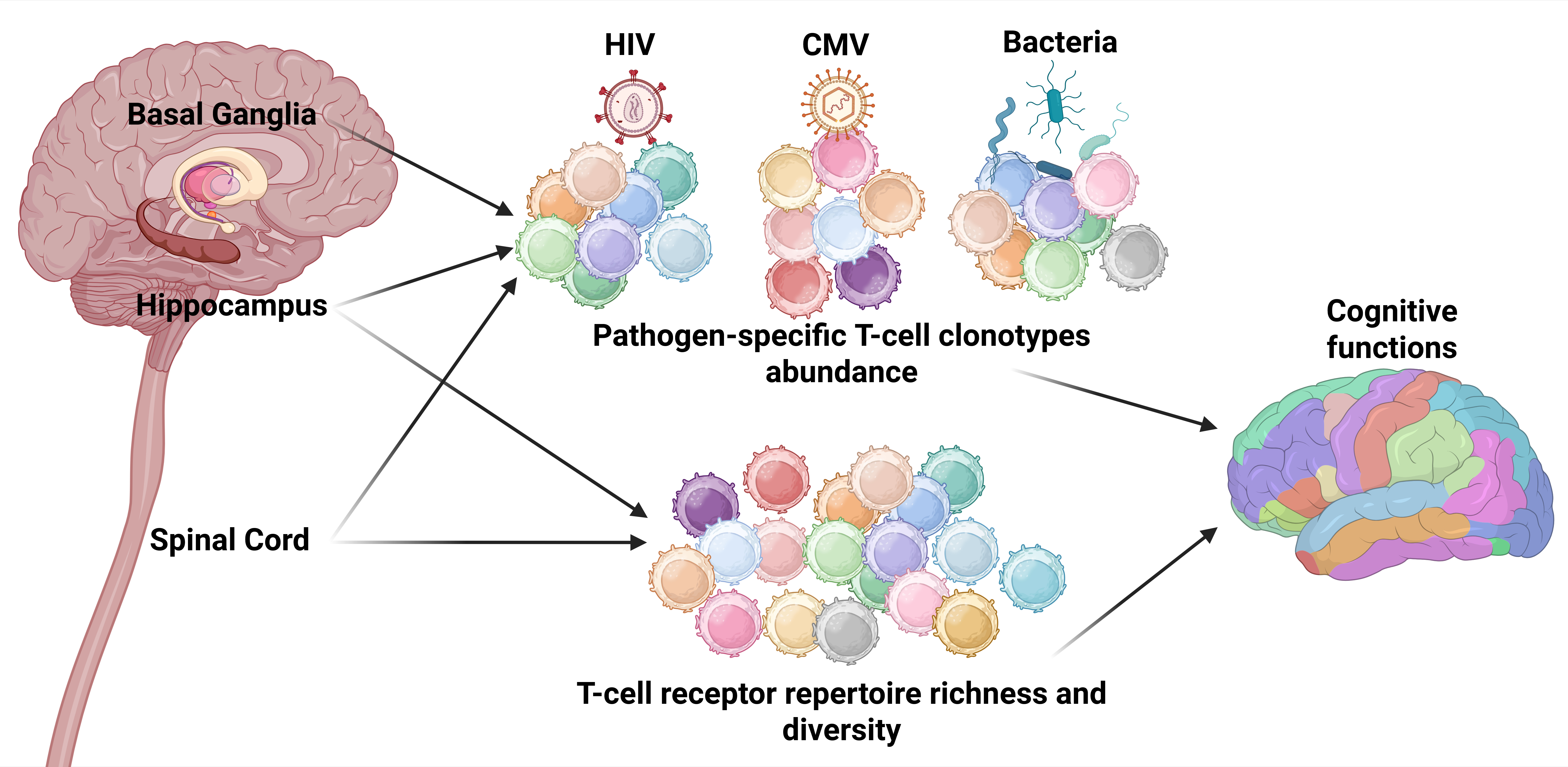

Supplement: S1 Fig — Conceptual overview of the study highlighting representative significant associations between T-cell receptor repertoire features, target epitopes, and cognitive outcomes across central nervous system tissues in people with HIV. (TIF) [file ppat.1014351.s005.tif]
