# Supplementary material for: Central Nervous System T-cell immune architecture, and not HIV burden, tracks with cognition under long-term viral suppression
Source: PLoS Pathog. 2026 Jun 15;22(6):e1014351. doi: 10.1371/journal.ppat.1014351 (PMC13286276; doi:10.1371/journal.ppat.1014351)
Supplement: Attachment — Submitted filename: PPATHOGENS-D-26-00422_reviewer comments.docx [file ppat.1014351.s007.docx]

**Central Nervous System T-cell immune architecture, 1 and not HIV burden, tracks with cognition under long-term viral suppression**

Mattia Trunfio1, Gemma Caballero1, Vanessa Gomez-Moreno1, Simon A. Mallal2,3, Celestine N. Wanjalla5,6, Angela Jones3, Karen Beeri3, Alan Wells1, Sarah LaMere1, Ben Gouaux4, Donald R. Franklin4, Michael Corley7, Ronald J. Ellis4, David J. Moore4, Scott L. Letendre1,4, Davey Smith1, Antoine Chaillon1*, Sara Gianella1*

**Comments for the Author:**

Trunfio et al. compared metrics of HIV viral persistence and T cell receptor repertoire (TCRR) in multiple central nervous system (CNS) sites to cognitive measurements prior to death in 12 patients. While HIV metrics did not correlate with cognitive performance, some TCRR metrics correlated with some cognitive measures, with generally more richness/diversity tracking with worse cognitive scores.

Overall, the article is well-written and figures are clear.

Even under highly effective anti-retroviral therapy, cogitative pathology still occurs. This study seeks to determine if there are links between infection, T cell receptor repertoire, and cognitive performance.

**MAJOR:**

**1:** A key aspect of the study is correlating measures of viral persistence and T cell receptor repertoire with cognitive performance scores. There will necessarily be a gap of time between biological measurements performed after the patient’s death and cognitive assessment prior to death. How stable were the cognitive metrics over time in this cohort? Alternatively, is there data on expected progression in a similar cohort? Was the length of time between biological measurements and cognitive assessment considered in any of the modeling?

**2:** Similarly, by necessity sampling of many CNS sites cannot be performed longitudinally and must be a snapshot of a particular time. How much variation in T cell receptor repertoire metrics is seen in sites that can be sampled over time (eg. PBMCs, cerebrospinal fluid) either in this cohort or in other published work, even from animal models if necessary?

**MINOR:**

**1:** Though this is an acknowledged limitation of the study, a biological mechanism for the observed correlations is lacking. Some method of assessing inflammation could significantly improve the link.

**2:** Do the multiple measures of the HIV reservoir correlate with each other in each tissue? For example, is more HIV DNA associated with more 2LTR, usGag, or msTat/Rev? If not, (for example a lot of DNA but not ms transcripts) does that suggest something about the amount of active viral replication in the particular tissue?

**3:** Line 121: “emerged as an important contributor…” How accurate is the term contributor vs. correlate. Is there direct evidence of contribution in all cited cases?

**4:** Line 140 cites perturbed and abnormal TCRR in PWH in peripheral blood. Is that the case in this study if the peripheral blood TCRR were compared to uninfected controls?

**5:** Line 176. First use of abbreviations in the results eg. FMC, HPC, usGAG, etc. should be defined. Figure legends do a good job of this, though.

**6:** Figure 5 radial diagrams. The % labels are hard to read, could use larger and/or darker font.

**7:** Line 504-505 Sentence beginning “Although substantial inter-host variability,” is lacking a word. Maybe, “Although there was substantial…”

**8:** Methods 3.4 for the TCR sequencing could use more detail on the IMMUNOVerse platform.
