# Supplementary material for: Central Nervous System T-cell immune architecture, and not HIV burden, tracks with cognition under long-term viral suppression
Source: PLoS Pathog. 2026 Jun 15;22(6):e1014351. doi: 10.1371/journal.ppat.1014351 (PMC13286276; doi:10.1371/journal.ppat.1014351)
Supplement: Attachment — Submitted filename: Response To Reviewers.docx [file ppat.1014351.s009.docx]

**Part I - Summary**

**Reviewer #1:** Trunfio and colleagues examined how HIV-1 DNA and TCR repertoires across distinct CNS regions relate to cognitive impairment in post-mortem tissues from ART-suppressed PWH, with cognitive scores assessed within a year of death. Using multivariate regression, they found that TCR diversity was highest in blood and largely compartmentalized across CNS anatomical sites. Notably, greater TCR diversity in the hippocampus and thoracic spinal cord associated with worse verbal and motor function, and regional HIV-specific clonotype frequencies also correlated with cognitive outcomes. There were no differences in HIV-1 DNA between sites (excluding the blood) nor associations to cognitive scores. Strengths of the study include the unique and comprehensive sampling opportunity of the Last Gift Cohort. All subjects had sustained ART suppression, which is a strength relative to similar studies that have been limited by cohort heterogeneity in viremic status. The novelty in this study is that to my knowledge, TCR repertoires across distinct regions of the brain in PWH have not been characterized in PWH. The major driving weaknesses are that it is devoid of inflammatory measurements, either soluble in the plasma or CNS or by transcriptomics. This is important because most of these patients fit the definition of immune non-responders (CD4<350) which are well known to exhibit persistently high levels of inflammation. A second weakness is that the associations between TCRRs and specific cognitive scores are not necessarily found within the brain regions that govern those neurological functions, leaving the implications unclear. The authors do touch on this caveat in the discussion in sampling TCR-high parenchymal versus TCR-low tissue subregions, however the authors would need spatial profiling to confirm this.

**AR:** We thank Reviewer1 for their time spent improving our manuscript, the appreciation, and the positive and helpful feedback.

We agree that the absence of direct inflammatory measurements (e.g., soluble markers or transcriptomic profiling) limits the ability to mechanistically link TCRR features to specific neuroinflammatory pathways. This is an important point, particularly in the context of individuals with incomplete immune reconstitution, who may exhibit persistent systemic and CNS immune activation. However, the primary objective of this study was not to directly quantify inflammation, but rather to characterize the tissue-embedded adaptive immune landscape across CNS compartments. In this framework, TCRR features should be interpreted as integrative and cumulative proxies of antigenic exposure and immune history over time, rather than direct measures of contemporaneous inflammatory states. Importantly, this distinction also reflects a complementary perspective to prior studies (e.g., PMID: 34108514; 34280844; 37112989), which have predominantly relied on soluble or transcriptomic markers capturing more proximal and dynamic immune processes. By contrast, TCRR provides insight into the longer-term, tissue-resident immunological architecture that may not be fully captured by these approaches (PMID: 32796830; 33732256). Accordingly, we interpret TCRR as a temporally integrated measure of neuroimmune history rather than a direct readout of active inflammatory pathways at the time of death. Cognitive assessment captures a relatively stable functional state during suppressive ART (at least in the few years preceding and following the assessment, e.g., PMID: 17914066; 25362201), whereas soluble inflammatory markers and transcriptomic profiles are highly dynamic and may be disproportionately influenced by acute perimortem factors, including terminal illness, cause of death, or transient inflammatory processes. In contrast, TCR repertoires reflect cumulative antigenic exposure and adaptive immune history over months to years, potentially making them more temporally aligned with cognitive status assessed prior to death. Prior studies have shown that dominant T-cell clonotypes and overall repertoire structure remain relatively stable over time, including in people with HIV on suppressive ART, despite ongoing immune turnover and antigenic stimulation [e.g., PMID: 33732256; PMID: 37949070]. **Similarly, cognitive trajectories in ART-suppressed PWH are often relatively stable over medium-short observation periods** (e.g., PMID: 17914066; PMID: 25362201). We have clarified this conceptual and temporal distinction, emphasizing that TCRR should be interpreted as an indirect proxy of neuroimmune activity. We also explicitly acknowledge that, particularly in a cohort with relatively low CD4+ T-cell counts and CD4/CD8 ratio <1 (now included for those participants with available data), persistent inflammation is likely present and may represent a key intermediary linking TCRR to cognitive outcomes.

We have revised the discussion to more explicitly acknowledge the lack of complementary biomarkers or direct measures of immune activity and to clarify the interpretative framework of TCRR metrics. We also emphasize that future studies integrating TCRR with paired inflammatory and transcriptomic data will be essential to delineate the mechanistic pathways linking immune activation to CNS injury in PWH. Detailed references to the corresponding revisions are provided in our responses to the specific comments of Rev#1 outlined in Part II.

Regarding the regional associations with cognitive domains, we agree that the anatomical regions in which associations were observed (hippocampus and thoracic spinal cord) do not correspond to the classical neuroanatomical substrates of the affected cognitive domains. We do not interpret these findings as evidence of region-specific functional localization. Instead, we interpret them within a framework of region-dependent neuroimmune interfaces. Regions such as the hippocampus and spinal cord are characterized by greater immune accessibility and proximity to CNS border compartments with immune trafficking (e.g., choroid plexus is adjacent to HPC, and TSC has a greater accessibility to meningeal and perivascular spaces and CSF circulation), and may therefore preferentially capture immune signals related to trafficking, antigen exposure, and immune activation. In this context, TCRR features measured in these regions are more likely to reflect integrated neuroimmune dynamics that may affect the whole CNS but can be detected more easily by bulk sampling of these regions compared to the other regions sampled for this study. rather than local neuronal function. In addition, bulk region-targeted sampling likely captured variable contributions from parenchymal, vascular, meningeal, and interface-associated immune compartments, which may differ across anatomical regions.

We have revised the discussion to more clearly articulate this interpretation and to avoid any implication of direct neuroanatomical mapping. Similarly, associations involving pathogen-specific clonotypes are now framed as reflecting tissue-contextualized immune signatures rather than direct functional effects of specific anatomical regions. Detailed references to the corresponding revisions are provided in our responses to the specific comments of Rev#1 outlined in Part II.

**Part II – Major Issues: Key Experiments Required for Acceptance**

**Reviewer #1:** - The authors find associations between regional T cell receptor repertoires and worse outcomes in specific neurocognitive measures such as verbal and motor functions (Figure 4); however, the significance of these findings is unclear because the specific regions in which these associations are observed — the hippocampus and thoracic spinal cord — do not primarily govern these functions. The hippocampus is mostly involved with memory, and the thoracic spinal cord primarily controls trunk muscle movement, not verbal or motor neurocognitive performance.
- Similarly, the authors observed CMV-specific clonotypes in the TSC to correlate with worse learning scores, however the TSC does not control learning.

**AR:** We agree that the anatomical regions in which associations were observed do not directly correspond to the classical neuroanatomical substrates of the affected cognitive domains. We do not interpret these findings as reflecting region-specific functional localization. Rather, we interpret them as signatures of region-specific neuroimmune interfaces. The hippocampus, due to its proximity to the choroid plexus, and the spinal cord, due to its accessibility to meningeal and perivascular compartments and integration with CSF circulation, are regions enriched in immune trafficking and border-associated immune activity. In this context, TCRR features measured in bulk tissue samples of these regions are more likely to reflect neuroimmune dynamics occurring at these interfaces which integrate systemic and CNS immune processes rather than strictly local neuronal function.

This interpretation is consistent with experimental evidence and animal models showing that T-cell–mediated immune activity at CNS interfaces (e.g., choroid plexus and leptomeninges) can influence cognitive and behavioral outcomes independently of strict anatomical localization of function [PMID: 20439540; 29731353; 23597431; 23335631].

We have revised the manuscript to explicitly clarify that these associations should not be interpreted as neuroanatomical mapping of cognitive functions, but rather as reflecting interface-driven neuroimmune processes. Specifically:

- Lines 449-488 of the revised manuscript, discussion: “Associations between TCRR and cognition emerged preferentially in specific regions such as the HPC and TSC. This selective signal may reflect differential sampling of tissue immunological niches. The HPC is a region where immune-mediated perturbation have been extensively characterized, given its high synaptic plasticity, dense microglial network, and susceptibility to inflammatory cytokines.[50,51] Experimental work has shown that T cell–derived signals can bidirectionally modulate hippocampal neurogenesis, synaptic remodeling, and cognitive functions, depending on the balance between regulatory and inflammatory pathways.[22,27,52,53] Moreover, HPC lies in close proximity to choroid plexus, a site of active immune surveillance enriched in T cells.[43,54] As a result, bulk hippocampal sampling may partially capture immune populations associated with border compartments, where T cell activity is richer and closely coupled to dynamic cognitive circuits, rather than exclusively reflecting parenchymal processes intrinsic to HPC function. In fact, these findings should not be interpreted as reflecting anatomical localization of cognitive functions to these regions. Similarly, compared with brain, TSC exhibits greater immune accessibility, higher relative contribution of meningeal and perivascular immune compartments, and closer integration with CSF circulation[55–58]. These features could potentially weight tissue sampling and bulk tissue TCRR profiling toward border-associated T-cell populations, where immune traffic and antigenic sampling are more pronounced. In contrast, cortical regions and BSG are characterized by tighter immune exclusion and lower T cell density,[43,54] potentially being less permissive to detectable TCRR–cognition coupling in bulk tissue analyses. Taken together, we interpret the apparent hippocampal and spinal specificity of the TCRR-cognition relationship as a reflection of T cells sampled at periphery-CNS interfaces, in line with prior evidence of T cells modulating neuroinflammation and cognitive functions from the leptomeninges and choroid plexus.[59–62] Overall, these associations should be interpreted as signatures of region-specific immune accessibility and interface dynamics, rather than as evidence of direct neuroanatomical mapping of cognitive functions. This framework explains why TCRR features measured in the HPC and TSC associated with cognitive functions that do not have a direct neuroanatomical substrate in these regions, and why higher TCRR richness in the TSC was associated with better recall, in contrast to its association with poorer performance in other domains. This divergence could be interpreted as the net effect of heterogeneous coexisting T-cell subpopulations captured by bulk TCRR profiling across anatomically and functionally distinct compartments (e.g., meningeal, perivascular, parenchymal), rather than as bidirectional effects of a single biological entity. The mechanistic interpretations proposed here, including increased immune cell trafficking, antigenic exposure, and compartment-specific immune activation, should be considered hypothesis-generating and not evidence of causality, as they cannot be directly validated in the absence of complementary cellular and molecular measurements. Future studies using spatial immunophenotyping, single-cell or single-nucleus transcriptomic profiling, complemented by vascular or barrier markers across regions, are warranted to verify such hypothesis, localize, and functionally characterize border-associated versus parenchymal T cells.”
- Lines 507-523 of the discussion, regarding epitope-specific clonotypes, cognitive domains and CNS regions: “As noted above, these associations should not be interpreted as reflecting direct functional roles of the sampled anatomical regions in the corresponding cognitive domains. We interpret them as region-dependent immune signatures shaped by differences in immune accessibility, antigen exposure, and T-cell trafficking across CNS compartments, collectively reflecting system-level neuroimmune processes. The abundance of epitope-specific clonotypes in each region may reflect where these cells are detectable, and potentially preferentially retained, at the time of sampling, rather than the site where their functional effects on cognition necessarily occur. For example, the association between higher abundance of HIV-specific clonotypes in the BSG and better processing speed and attention/working memory is unlikely to indicate a region-specific functional effect. Instead, it may reflect more effective anti-HIV immune surveillance at the CNS level, with HIV-specific T cells more easily detectable, accumulating or persisting in subcortical regions, that have historically been implicated as preferential sites with significant antigenic exposure in untreated infection and vulnerability to HIV-related injury.[66,67] Within this framework, the BSG may represent a site of enhanced detectability of these clonotypes due to local antigenic cues, whereas their relationship with neurocognitive outcomes may reflect distributed or system-level neuroimmune dynamics.”
- In this regard, we have also revised Figure 4 to more accurately reflect the sampling context. Specifically, we added intermediate schematic panels illustrating representative neuroimmune interfaces in the hippocampus (including proximity to the choroid plexus) and spinal cord (including meningeal and CSF-associated compartments) and clarified in the legend that these regions capture mixed anatomical and immune compartments rather than discrete structures. We have also revised the figure legend to explicitly state that immune signals detected in these regions likely reflect this region-dependent sampling of neuroimmune interfaces rather than precise anatomical localization, and we clarified that, although neuroimmune interfaces are present throughout the CNS, their relative contribution to bulk tissue sampling and immune signal detection is not uniform across regions, and is likely greater in areas such as the hippocampus and spinal cord compared with more immune-restricted regions. Figure 4 legend addition: “Middle panels illustrate region-dependent neuroimmune interfaces and bulk sampling context for the HPC and TSC. Region-targeted bulk tissue sampling may include a mixture of parenchymal (gray and white matter), perivascular, and meningeal/interface compartments, with variable contributions across regions. Although meningeal and perivascular interfaces are present throughout the CNS, their relative contribution to immune signal detection may be region-dependent, being greater in areas such as the HPC (proximity to choroid plexus) and SC (greater meningeal and CSF interface) compared with more immune-restricted regions (OCC, FMC, BSG). Accordingly, associations should not be interpreted as reflecting direct neuroanatomical localization of cognitive functions, but rather as reflecting interface-biased neuroimmune signals captured through region-specific sampling context”.
- We also note that revisions made in response to related reviewer comments, particularly regarding the use of bulk region-targeted tissue without separation of gray and white matter compartments and the inability to distinguish CD4+ from CD8+ T-cell subsets, further reinforce this interpretive framework and clarify that the observed associations reflect composite neuroimmune signals rather than discrete region-specific effects.

- Do the authors have CD8 T cell counts as well? Many of these subjects likely have inverted CD4/CD8 ratios, and it would be helpful to know whether TCRs are more reflective of CD4 versus CD8 repertoires.

**AR:** Because TCRR profiling was performed on bulk tissue, the observed repertoires reflect the combined contribution of CD4+ and CD8+ T cells. Peripheral CD8+ T-cell counts and CD4/CD8 ratios were available only in a subset of participants (n=7), all of whom had inverted CD4/CD8 ratios, consistent with persistent immune dysregulation despite long-term viral suppression. However, the limited availability of these data precluded formal inclusion in multivariable models without substantial loss of statistical power.

- In the result sections describing the study population, lines 167-169, we added “All participants with available data of CD8+ T-cells (n=7) had a CD4/CD8 ratio <1, consistent with persistent immune dysregulation despite long-term viral suppression: median CD8^+^ T-cell count and CD4/CD8 ratio were 278 (209-366) cells/μL and 0.7 (0.6-0.9).”

We therefore cannot determine whether the observed TCRR features are preferentially driven by CD4+ or CD8+ T-cell populations. Given the known expansion and persistence of antigen-experienced CD8+ T cells in treated HIV infection, it is plausible that CD8+ T cells contribute substantially to the observed repertoire signals; however, this cannot be directly assessed in the current study. In addition, peripheral CD4/CD8 measurements may not accurately reflect the composition of CNS-infiltrating T-cell populations. We have clarified these limitations in the manuscript and now explicitly acknowledge that subset-specific contributions cannot be resolved. Future studies using subset-resolved or single-cell approaches will be necessary to address this question.

- In the discussion, lines 593-598, section about limitations: “For the same reason, we could not distinguish the relative contribution of CD4+ and CD8+ T-cell populations to the observed repertoire features. Peripheral CD8+ T-cell counts and CD4/CD8 ratios were available only in a subset of participants, precluding their inclusion in the models without substantial loss of power. As such, the subset-specific drivers of the observed TCRR associations, including the relative contribution of cytotoxic versus regulatory immune components, cannot be determined.”

- The association between higher TCR diversity and worse neurocognitive outcomes is somewhat contrary to the broader literature. Oligoclonality or skewed repertoire diversity — rather than expanded diversity — has been associated with neurocognitive impairment. The significance of this finding and the potential mechanism underlying it is somewhat uncertain.

**AR:** We agree that the direction of this association appears to contrast with prior literature, where reduced TCRR diversity or oligoclonality has been associated with adverse outcomes. However, these observations derive from peripheral blood and may not translate to the CNS, where immune composition, antigen exposure, and compartmental constraints differ fundamentally. Furthermore, studies linking TCRR architecture in peripheral blood to neurocognitive outcomes have been conducted in populations without HIV and for other neurological conditions (e.g., neurodegenerative dementias, multiple sclerosis, neuropsychiatric conditions), further limiting comparability. In PWH, Turner et al (PMID 33732256) showed that, in peripheral blood of PWH, higher TCRR diversity is associated with increased expression of T cell activation and IFN-γ–associated genes, which, if it is the same in the CNS, could be detrimental. We have revised the discussion to explicitly frame this finding as a compartment-specific relationship. In the CNS, higher TCRR richness and diversity may reflect increased permeability of neuroimmune interfaces, enhanced T-cell recruitment, broader antigenic exposure, higher microbial burden, and/or dysregulated neuroimmune interactions, rather than preserved immune homeostasis. Accordingly, interpretations derived from peripheral blood, including our initial hypothesis, may not directly apply to CNS immune environments.

- Third paragraph of the discussion, lines 409-441: “Contrary to our hypothesis, higher TCRR richness and diversity within the CNS were associated with poorer cognitive performance across multiple domains, except for higher richness in the TSC associating with better recall. The direction of this association appears to contrast with prior literature in population without HIV (e.g., Alzheimer’s disease),[17,21] where reduced diversity or oligoclonality has been associated with worse neurological features. However, these prior observations derive primarily from peripheral blood and may not translate to the CNS, where immune composition, antigen exposure, and compartmental constraints differ fundamentally. In HIV and other chronic viral infections, prior studies showed clonal expansions, repertoire skewing, and reduced breadth,[28–30,42] thereby we anticipated that lower richness and diversity would be associated with worse cognition, whereas broader repertoires would reflect better preserved immune homeostasis. However, also these studies were performed in peripheral blood.[28–30,42] In the CNS, that is normally tightly regulated and poor in T cells [43], higher TCRR richness and diversity may reflect increased permeability of neuroimmune interfaces, enhanced T-cell recruitment and retention, broader antigenic exposure, and ultimately “immune crowding”[44]. Because we could not quantify immune cell density or perform phenotypic characterization, we cannot determine whether increased richness reflects a higher number of infiltrating T cells, a broader distribution of clonotypes within a stable population, or both. Complementarily, higher TCRR richness and diversity in the CNS may reflect also “antigenic overload” due to impaired antigenic clearance, higher burden of co-infections, increased microbial translocation,[40,45] or dysregulated responses to self-antigens. All these carry detrimental consequences for neuronal and glial function, and have been previously linked to cognitive impairment.[8,40,46,47] This interpretation is also consistent with other evidence in PWH that found higher TCRR diversity in the blood to be associated with increased expression of T cell activation and IFN-γ–associated genes,[30] suggesting a link with heightened immune activation. Thus, in ART-suppressed PWH, higher CNS TCR richness and diversity may reflect neuroimmune activity associated with poorer cognitive outcomes. Because we lack normative reference ranges for TCRR metrics and comparison groups of people without HIV and PWH off ART, we cannot determine whether the observed values reflect normal variation or a fundamentally altered state. Nevertheless, the observed associations indicate that variation within the range observed in PWH on suppressive ART remains biologically meaningful and tracks inter-individual cognitive differences. Taken together, these findings suggest that the TCRR-cognition signal may be compartment-specific, and that hypotheses derived from peripheral blood may not directly apply to the CNS”.

- The positive association between HIV-specific T cell clonotype frequency and better GDS and processing speed scores is intriguing but difficult to interpret. One possibility is that a higher proportion of HIV-specific clonotypes reflects more active immune surveillance of the tissue. However, neither the HIV-specific clonotype frequency nor the neurocognitive outcomes correlated with HIV DNA levels in these regions, leaving the biological significance of this association uncertain.

**AR:** We agree that the lack of association between HIV-specific clonotypes, HIV reservoir measures, and cognitive outcomes complicates interpretation. However, this dissociation may indicate that antigen-specific T-cell responses capture aspects of immune surveillance or control that are not fully reflected by the bulk virological measures used in this study. In this framework, immune composition and antigen-specific responses may be more relevant to neurocognitive outcomes than reservoir size alone. While we also quantified HIV transcriptional activity (unspliced Gag RNA and multiply spliced Tat/Rev RNA), these measurements represent bulk, cross-sectional estimates of viral transcription at the time of sampling and may not fully capture the fraction of virus that is immunologically relevant. Low-level or spatially restricted transcription, intermittent antigen expression, or prior antigenic exposure may not be adequately reflected by these measures, especially in the context of suppressive ART. In addition, total HIV DNA does not distinguish between intact and defective proviruses, and even transcriptional readouts may not resolve which fraction of the reservoir can produce antigen sufficient to drive or maintain T-cell responses. As such, the absence of correlation with these measures should not be interpreted as evidence against a role of HIV-specific immune responses.

We have revised the discussion to clarify that different dimensions of viral persistence, including intact provirus (e.g., via IPDA), inducible or translation-competent reservoirs, and temporally dynamic transcriptional activity, may relate differently to antigen-specific immune responses, but cannot be resolved within the constraints of the current study.

- End of the paragraph regarding HIV reservoir discussion, lines 383-408: **“**Altogether, these findings suggest that static quantitative measures of HIV persistence and activity may have limited explanatory value for cognitive outcomes under long-term viral suppression. In this context, proviral burden and snapshot measures of residual viral transcription may be insufficient to induce CNS injury detectable at the clinical level. Alternatively, total HIV DNA and bulk measures of HIV persistence may not fully capture the fraction of virus that is immunologically active or relevant for CNS injury. While we quantified usGag and msTat/Rev RNA, these bulk measurements reflect transcriptional activity at the time of death and may not adequately capture low-level, spatially restricted, or intermittent antigen expression that is most relevant to immune recognition under suppressive ART. Consistent with this, a similar pattern was observed across CNS tissues, with total HIV DNA associating with usGag but not with msTat/Rev RNA, and early and late transcriptional markers largely uncoupled, indicating that initiation of transcription may relate to reservoir size, whereas progression to later transcriptional stages is not sustained under suppressive ART. As such, total HIV DNA and RNA-based measures may not reflect the antigenic stimuli driving local T-cell responses, potentially explaining the dissociation between HIV reservoir measures and TCRR features observed here. Furthermore, comparable levels of proviral persistence and activity may be associated with markedly different degrees of immune activation across individual, as several factors (e.g., comorbidities, medications, co-infections, and genetic differences) shape their relationship.[40,41] Lastly, although both the HIV reservoir and TCRR are shaped by infection history and tissue context, TCRR architecture reflects the composition and distribution of local T cell responses and integrates cumulative antigenic exposure, which may provide a complementary and temporally integrated readout of the neuroimmune environment and longer-term immune dynamics. Future studies integrating measures of intact and inducible reservoir with temporally resolved immune profiling (e.g., transcriptomics) will be necessary to better resolve the relationship between viral persistence, HIV-specific immune responses, and CNS outcomes.”
- In the discussion paragraph on epitope-specific clonotypes, lines 494-507: “in the BSG, a greater abundance of HIV Gag-specific T-cell clonotypes was associated with better global cognition, processing speed, and attention/working memory, independently of HIV reservoir. This dissociation suggests that the presence of HIV-specific clonotypes may reflect immune surveillance or containment mechanisms that are not directly captured by bulk measures of viral burden, which include both intact and defective proviruses. Even transcriptional metrics may not capture the fraction of virus that is antigenically relevant, as low-level or intermittent expression to sustain T-cell responses may not be detected in cross-sectional measurements. These T cells may represent effective immune surveillance or containment, potentially limiting inflammation, HIV activity, and local injury, consistent with prior evidence of effective HIV control by Gag-specific CD4+ and CD8+ T cells [63–65]. The dissociation between these clonotypes and HIV reservoir further supports a shift away from a reservoir-centric model of HIV-associated neuro-injury toward models emphasizing immune composition, antigenic history, co-infections, and surveillance dynamics under long-term viral suppression.”

- Lines 395-397 regarding the underlying nature of the high TCR richness. I do agree that leukocytosis in the CNS could plausibly explain the findings. It is unfortunate that the authors do not have the cellular or transcriptomic data to test this hypothesis.

**AR:** We agree that the interpretation of higher TCR richness as reflecting increased immune cell infiltration or leukocytosis within the CNS remains speculative in the absence of supporting cellular or molecular data. In particular, we did not have access to complementary measures of tissue immune cell density, phenotypic characterization, or transcriptomic and cytokine profiling that would allow us to directly assess immune infiltration, activation state, or local inflammatory tone. As such, we have revised the discussion to explicitly frame this as one of several plausible interpretations rather than an inferred mechanism. We also note that increased TCRR richness could alternatively reflect broader antigenic exposure, altered trafficking dynamics, or compartmental retention of diverse T-cell populations, which cannot be disentangled in the current dataset. We now more clearly acknowledge that integrated cellular, spatial, and transcriptomic approaches will be required to distinguish between these possibilities.

- Discussion, third paragraph, lines 420-441: “In the CNS, that is normally tightly regulated and poor in T cells [43], higher TCRR richness and diversity may reflect increased permeability of neuroimmune interfaces, enhanced T-cell recruitment and retention, broader antigenic exposure, and ultimately “immune crowding”[44]. Because we could not quantify immune cell density or perform phenotypic characterization, we cannot determine whether increased richness reflects a higher number of infiltrating T cells, a broader distribution of clonotypes within a stable population, or both. Complementarily, higher TCRR richness and diversity in the CNS may reflect also “antigenic overload” due to impaired antigenic clearance, higher burden of co-infections, increased microbial translocation,[40,45] or dysregulated responses to self-antigens. All these carry detrimental consequences for neuronal and glial function, and have been previously linked to cognitive impairment.[8,40,46,47] This interpretation is also consistent with other evidence in PWH that found higher TCRR diversity in the blood to be associated with increased expression of T cell activation and IFN-γ–associated genes,[30] suggesting a link with heightened immune activation. Thus, in ART-suppressed PWH, higher CNS TCR richness and diversity may reflect neuroimmune activity associated with poorer cognitive outcomes. Because we lack normative reference ranges for TCRR metrics and comparison groups of people without HIV and PWH off ART, we cannot determine whether the observed values reflect normal variation or a fundamentally altered state. Nevertheless, the observed associations indicate that variation within the range observed in PWH on suppressive ART remains biologically meaningful and tracks inter-individual cognitive differences. Taken together, these findings suggest that the TCRR-cognition signal may be compartment-specific, and that hypotheses derived from peripheral blood may not directly apply to the CNS.”
- Discussion, limitations section: “A second important limitation is the absence of soluble or transcriptomic inflammatory measurements. As a result, we cannot directly link TCRR features to specific inflammatory pathways or quantify the degree of neuroinflammation within each tissue. However, inflammatory markers are highly dynamic and may be influenced by perimortem factors, including terminal illness and cause of death, whereas cognitive assessments were performed months prior to death and appeared on average stable over at least the last 2 years of life. In this context, TCRR architecture may be more temporally aligned with cognition than highly dynamic inflammatory markers and it may provide a more integrative readout of neuroimmune activity, but mechanistic interpretation remains indirect and without defined immune mediators and pathways. Future studies combining TCRR profiling with cytokine measurements, spatial transcriptomics, and single-cell approaches will be necessary to resolve these relationships.”

**Part III – Minor Issues: Editorial and Data Presentation Modifications**

**Reviewer #1:** - Minor: it would improve readability if the acronyms were also spelled out in the text, when they are first referred to.

**AR:** Apologies, we have revised the manuscript to define all acronyms at their first use.

- Minor: lines 207-212, the authors should refer to the specific figure legend in the text.

**AR:** We have added explicit figure references in the relevant sections to improve readability.

**Reviewer #2:** Trunfio et al. compared metrics of HIV viral persistence and T cell receptor repertoire (TCRR) in multiple central nervous system (CNS) sites to cognitive measurements prior to death in 12 patients. While HIV metrics did not correlate with cognitive performance, some TCRR metrics correlated with some cognitive measures, with generally more richness/diversity tracking with worse cognitive scores.
Overall, the article is well-written and figures are clear.
Even under highly effective anti-retroviral therapy, cogitative pathology still occurs. This study seeks to determine if there are links between infection, T cell receptor repertoire, and cognitive performance.
A particular strength is the samples assessed as CNS tissue from people with HIV is relatively rare.

**AR:** We thank the reviewer for the careful evaluation of our manuscript and for the positive and constructive feedback. We appreciate the recognition of the strengths of our study, including the use of rare, well-characterized CNS tissue samples from people with HIV and the clarity of the presentation. We are pleased that the overall rationale, analysis, and figures were considered clear and well-written.

**Reviewer #2:** 1: A key aspect of the study is correlating measures of viral persistence and T cell receptor repertoire with cognitive performance scores. There will necessarily be a gap of time between biological measurements performed after the patient’s death and cognitive assessment prior to death. How stable were the cognitive metrics over time in this cohort? Alternatively, is there data on expected progression in a similar cohort? Was the length of time between biological measurements and cognitive assessment considered in any of the modeling?

**AR:** We agree that the temporal gap between antemortem cognitive assessment and post-mortem biological measurements is an important consideration. In our cohort, the final cognitive assessment was performed relatively close to death (median 8 months). We initially performed sensitivity analyses adjusting for the interval between cognitive assessment and death, observing no meaningful change in the results. Due to the limited number of covariates that could be included in the model and the limited variability in the range of such a covariate we did not report this analysis in the original manuscript. We have now added a sentence that state that adjusting for the interval between cognitive assessment and death did not affect the observed associations.

- Results for both TCRR metrics-cognitive scores and epitope-specific T clonotypes-cognitive scores, we added: “In sensitivity analyses including the interval between cognitive assessment and autopsy as a covariate, the magnitude and direction of the observed associations were unchanged.”
- Methods: “Sensitivity analyses additionally adjusted for the interval between final cognitive assessment and death to evaluate whether temporal separation between cognitive testing and post-mortem tissue sampling influenced the observed associations.”

Prior neurocognitive assessments were available for 8 participants, at a median time of 20 months (IQR 15; 33) before the assessment used in the present study. Across these participants, the median change in GDS was 0.28 (IQR −0.06; 0.53). Two participants transitioned from normal to impaired performance, whereas the remaining participants stayed within the same category, including two who improved GDS staying within the normal performance category, and four persistently impaired with worsening GDS. After normalizing for time between assessments, the median change in GDS was +0.01/month (IQR −0.004 to +0.02), corresponding to approximately +0.12/year. Thus, although some quantitative worsening occurred, global impairment status was largely stable over the available pre-autopsy interval (almost 2 years). This is in line with prior studies showing relatively stable cognitive trajectories over medium-short observation times in PWH (PMID: 17914066; 25362201). We have added these descriptive data to the results and clarified in the discussion that cognitive performance was not uniformly static but showed limited categorical change in the available longitudinal subset.

- Methods, lines 676-682: “The interval between the neurocognitive assessment and death was calculated for each participant. For participants with more than one neurocognitive assessment available, we also calculated change in global and domain deficit scores between the final and the preceding assessment, normalized by the interval between assessments, for descriptive purposes of the cognitive trajectory.”
- End of the first chapter of the results, lines 174-183: **“**Prior neurocognitive assessments were available for 8 participants, performed a median of 20 (15–33) months before the assessment used in this study. Among these, the median change in GDS between the prior and final assessment was 0.28 (−0.06 to 0.53). Two participants transitioned from normal to impaired performance, whereas the remaining participants stayed within the same category, including two who improved GDS staying below the 0.5 GDS cut-off, and four persistently impaired with worsening GDS. After normalizing for time between assessments, the median change in GDS was +0.01/month (IQR −0.004 to +0.02), corresponding to approximately +0.12/year. Thus, although some quantitative worsening occurred, global impairment status was largely stable over the available pre-autopsy interval.**”**
- Discussion, limitations section, lines 599-620: “the cross-sectional design precludes determination of the temporal sequence between TCRR and cognition: we cannot establish whether the observed TCRR features preceded cognitive impairment, contributed to its development, or instead reflect persistent immune surveillance or adaptation following prior CNS injury. Thus, some of the observed associations may represent immune responses to historical or cumulative damage rather than drivers of ongoing pathology, or both. This limitation is partially mitigated by the nature of TCRR itself: it reflects cumulative antigenic exposure and long-term immune dynamics. Longitudinal studies in peripheral blood indicate that TCRR architecture evolves gradually over years to decades, largely driven by aging and chronic antigen exposure[81]. Over shorter timeframes (months to one year), dominant clonotypes and overall repertoire structure appear relatively stable[82], despite ongoing antigen-driven fluctuations or HIV infection[30,83]. Unlike soluble inflammatory markers, which can shift over hours to days, the clonal composition of T cell populations, especially in an immunologically privileged compartment like the CNS, reflects cumulative antigen exposure and clonal selection over months to years. Significant reshaping of the CNS TCR repertoire would require sustained antigenic drive or major immune reconstitution events, neither of which is expected in ART-suppressed individuals over short timeframes; however, longitudinal data in CNS tissues are lacking. In this context, although TCRR was measured at a single time point, its short-term stability in peripheral compartments, together with the limited change in cognitive status in our cohort, in line with prior longitudinal studies showing relative stability of cognitive trajectories over few years in PWH[84,85], support the plausibility of biologically meaningful associations within this timeframe, although causal relationships cannot be inferred”

2: Similarly, by necessity sampling of many CNS sites cannot be performed longitudinally and must be a snapshot of a particular time. How much variation in T cell receptor repertoire metrics is seen in sites that can be sampled over time (eg. PBMCs, cerebrospinal fluid) either in this cohort or in other published work, even from animal models if necessary?
In both cases, any additional data or references could strengthen the paper.

**AR:** We agree that TCRR profiling in this study represents a single time point and cannot capture longitudinal dynamics within CNS tissues. Importantly, longitudinal data on TCRR dynamics in CNS compartments are currently lacking, both in people with HIV and in other populations. However, available evidence from peripheral blood suggests that while TCRR composition evolves gradually over years to decades, primarily driven by aging and chronic antigen exposure, and with more pronounced changes in CD8⁺ than CD4⁺ compartments (PMID: [37515916](https://pubmed.ncbi.nlm.nih.gov/37515916/); [35708913](https://pubmed.ncbi.nlm.nih.gov/35708913/)), dominant clonotypes and overall repertoire structure tend to remain relatively stable over shorter intervals (months to one year; PMID [31226930](https://pubmed.ncbi.nlm.nih.gov/31226930/)). In the context of chronic HIV and long-term ART, persistent clonal expansions and partial stabilization of repertoire architecture after years of ART have been described (PMID [33732256](https://pubmed.ncbi.nlm.nih.gov/33732256/); 37949070). In our study, cognitive assessments were performed relatively close to death (median 8 months), and additional longitudinal data in a subset of participants showed limited categorical change in cognitive status over the preceding interval. In this context, the observed associations between TCRR features and cognition are less likely to reflect transient immune fluctuations and more likely to represent integrated immune states that persist over clinically relevant timeframes. We have revised the discussion to clarify that TCRR should be interpreted as a timepoint-specific but biologically integrated measure, while also acknowledging that its temporal dynamics in CNS tissues remain incompletely defined.

- Same paragraph of the discussion, lines 599-620 as per previous comment.

**Reviewer #2:** 1: Though this is an acknowledged limitation of the study, a biological mechanism for the observed correlations is lacking. Some method of assessing inflammation could significantly improve the link.

**AR:** We agree that mechanistic insight is limited by the absence of inflammatory or transcriptomic data, and that such measurements could provide a more direct link between immune activity and cognitive outcomes. We have revised the discussion to more clearly frame our interpretations as hypothesis-generating rather than causal. At the same time, we note that soluble inflammatory mediators and transcriptomic profiles are highly dynamic and may be particularly susceptible to perimortem confounding, including terminal illness, cause of death, and acute physiological perturbations. In contrast, TCR repertoire architecture reflects cumulative antigenic exposure and longer-term immune dynamics, potentially providing a more temporally integrated readout of neuroimmune history in a study design where cognitive assessments preceded death. Accordingly, rather than attempting to infer direct causal mechanisms, we interpret our findings within a framework of biologically plausible pathways, such as antigen-driven immune recruitment, chronic immune activation, and compartment-specific immune surveillance, while explicitly acknowledging that these cannot be directly tested in the absence of complementary molecular data. We have strengthened the discussion to clarify these points and to emphasize the need for integrated multi-omic and spatial approaches to resolve underlying mechanisms.

- Lines 481-488, discussion: “The mechanistic interpretations proposed here, including increased immune cell trafficking, antigenic exposure, and compartment-specific immune activation, should be considered hypothesis-generating and not evidence of causality, as they cannot be directly validated in the absence of complementary cellular and molecular measurements. Future studies using spatial immunophenotyping, single-cell or single-nucleus transcriptomic profiling, complemented by vascular or barrier markers across regions, are warranted to verify such hypothesis, localize, and functionally characterize border-associated versus parenchymal T cells.”

2: Do the multiple measures of the HIV reservoir correlate with each other in each tissue? For example, is more HIV DNA associated with more 2LTR, usGag, or msTat/Rev? If not, (for example a lot of DNA but not ms transcripts) does that suggest something about the amount of active viral replication in the particular tissue?

**AR:** We thank the reviewer for this suggestion. We examined correlations among HIV reservoir measures within each tissue. In all CNS tissues, total HIV DNA was positively associated with unspliced Gag HIV RNA (in the table below the results of correlations within each CNS tissue), but not with multiply spliced Tat/Rev RNA. UsGag and msTat/Rev were not correlated with each other. No correlations were observed in PBMCs.

| FMC | BSG | HPC | OCC | TSC |
| --- | --- | --- | --- | --- |
| ρ 0.57, p=0.053 | ρ 0.64 p=0.024 | ρ 0.70, p=0.011  (and usGag with Tat/Rev ρ 0.72, p=0.0077)- | ρ 0.65, p=0.021 | ρ 0.55, p=0.079 |

This pattern suggests a partial coupling between reservoir size and early transcriptional activity, but a decoupling from later stages of viral transcription. These findings are consistent with the concept that, under suppressive ART, HIV transcription is frequently initiated but does not progress to fully productive transcription, reflecting transcriptional blocks, defective proviruses, or regulatory constraints. Together, these observations support the interpretation that different reservoir measures capture distinct biological dimensions of HIV persistence, and that bulk measures of proviral DNA may not reflect the fraction of virus that is transcriptionally active or immunologically relevant. We have added these analyses and interpretations to the results and discussion.

- End of the second chapter of the Results: “In all CNS regions, total HIV DNA correlated with _us_Gag but not with _ms_Tat/Rev RNA nor with 2LTR DNA, and no consistent relationships were observed between early and late transcripts (**S2 Table**).” And we add the previous table as supplementary table 2.
- Discussion on reservoir findings, lines 387-398: “Alternatively, total HIV DNA and bulk measures of HIV persistence may not fully capture the fraction of virus that is immunologically active or relevant for CNS injury. While we quantified usGag and msTat/Rev RNA, these bulk measurements reflect transcriptional activity at the time of death and may not adequately capture low-level, spatially restricted, or intermittent antigen expression that is most relevant to immune recognition under suppressive ART. Consistent with this, a similar pattern was observed across CNS tissues, with total HIV DNA associating with usGag but not with msTat/Rev RNA, and early and late transcriptional markers largely uncoupled, indicating that initiation of transcription may relate to reservoir size, whereas progression to later transcriptional stages is not sustained under suppressive ART. As such, total HIV DNA and RNA-based measures may not reflect the antigenic stimuli driving local T-cell responses, potentially explaining the dissociation between HIV reservoir measures and TCRR features observed here.”

3: Line 121: “emerged as an important contributor…” How accurate is the term contributor vs. correlate. Is there direct evidence of contribution in all cited cases?

**AR:** We agree and have revised the wording from “contributor” to “associate” or “correlate” where appropriate to avoid overinterpretation.

4: Line 140 cites perturbed and abnormal TCRR in PWH in peripheral blood. Is that the case in this study if the peripheral blood TCRR were compared to uninfected controls?

**AR:** We agree that we do not include an HIV-uninfected control group in this study and therefore cannot directly assess whether TCRR features in peripheral blood are perturbed relative to uninfected individuals. Statements regarding altered TCRR in people with HIV are based on prior literature rather than on direct comparisons within our dataset. We have revised the manuscript to clarify this point and to emphasize that our analyses focus on within-cohort variation. We have also explicitly acknowledged the absence of HIV-uninfected control groups as a limitation when interpreting TCRR features.

- Introduction **“**In PWH, TCRR perturbations arise early and persist despite effective viral suppression.[28–31] Off ART, high viral loads are associated with reduced repertoire diversity, oligoclonality, and other TCRR abnormalities in peripheral blood.[28–31] Although ART decreases HIV-specific clonal expansions, persistent TCRR perturbations remain, and correlate with immune activation and T-cell exhaustion.[30] Despite this, the contribution of TCRR alterations to the brain and mental health of PWH remains largely unexplored, data on TCRR perturbations in tissues other than blood are scarce,[32] and no prior studies have examined how the regional CNS TCRR architecture and HIV reservoir jointly relate to cognition.**”**
- Discussion, lines 435-441: “Because we lack normative reference ranges for TCRR metrics and comparison groups of people without HIV and PWH off ART, we cannot determine whether the observed values reflect normal variation or a fundamentally altered state. Nevertheless, the observed associations indicate that variation within the range observed in PWH on suppressive ART remains biologically meaningful and tracks inter-individual cognitive differences.”

5: Line 176. First use of abbreviations in the results eg. FMC, HPC, usGAG, etc. should be defined. Figure legends do a good job of this, though.

**AR:** Apologies, we have revised the manuscript to correct this

6: Figure 5 radial diagrams. The % labels are hard to read, could use larger and/or darker font.

**AR:** We have increased font size and contrast in Figure 5 to improve readability.

7: Line 504-505 Sentence beginning “Although substantial inter-host variability,” is lacking a word. Maybe, “Although there was substantial…”
**AR:** Corrected.

8: Methods 3.4 for the TCR sequencing could use more detail on the IMMUNOVerse platform.

**AR:** We thank the reviewer for this comment. We have revised the relative methods section to provide additional methodological detail on the IMMUNOVerse platform and to improve the precision of our terminology. The revised text now specifies that TCR repertoire libraries were prepared using the Archer Immunoverse-HS TCR reagents for Illumina NovaSeq6000 (P/N dSK0159). We also clarify that the assay uses Anchored Multiplex PCR with panel-specific unidirectional gene-specific primers, molecular barcode adapters, and nested PCR to generate target-enriched libraries for Illumina sequencing. In addition, we revised the wording from “paired α and β chains” to “TCRR” to avoid implying native chain pairing in this bulk sequencing approach, and we clarified the downstream MiXCR and immunarch analysis pipeline, including restriction to productive in-frame rearrangements.

- Methods, lines 697-707: “Bulk high-throughput sequencing of TCRR was performed on bulk CNS tissues and matched PBMCs using the Archer Immunoverse-HS TCR reagents for Illumina (P/N dSK0159; NovaSeq 6000) on the IMMUNOVerse® platform (Vanderbilt University Medical Center). Because tissues were not sorted into T-cell subsets, TCRR profiles reflect the combined contribution of CD4+ and CD8+ T cells. Library preparation followed the Archer Immunoverse-HS TCR protocol for Illumina and used Anchored Multiplex PCR with panel-specific unidirectional gene-specific primeres, molecular barcode adapters, and nested PCR to generate target-enriched libraries for Illumina sequencing. Resulting FASTQ files underwent standardized quality control and annotation using MiXCR[39] and analyzed with the Immunarch R package (v0.10.3)[40] for CDR3 extraction, clonotype assembly, identification of unique productive rearrangements, and computation of TCRR metrics. Analyses were restricted to in-frame productive rearrangements.”

**Summary Reviewer #3:** This is an exhaustive and comprehensive study exploring HAND pathogenesis in the context of ART using precious and very rare autopsy samples from the Last Gift Program. The authors have used state of the art methods and analyses. The weaknesses are detailed in the manuscript including the small number of brains but two exceptionally important issues are not discussed:
* the tissues that were analysed did not include deep white matter. The methods used bulk tissue with the authors explaining that there would be some adjacent tissue ie white matter but the precise neuropathology of HAND is not understood. Certainly in the past it was more of a deep white matter pathology involving especially the frontal white matter and part of the basal ganglia but not all of the basal ganglia. It would have been far better to have included both cortical tissue and deep white matter.
*the second issue relates to the cognitive impairment - it is not at all clear when this developed: we know when it was tested for but not its onset and progression. This is fundamental to understanding pathogenesis as at least some of the deficits likely reflected past damage with an appropriate current TCRR response now that has controlled the limited replication of whole virus or part thereof. This then changes the whole new suggested paradigm of pathogenesis and makes it only a possibility rather than a certainty.

**Part II – Major Reviewer #3:** Please see above - white matter needs to be obtained and more precise details for the cognitive impairment

**AR:** We thank the reviewer for the careful evaluation of our manuscript and for highlighting these important points. We appreciate the recognition of the uniqueness of the Last Gift cohort and the comprehensive nature of the analyses.

We thank the reviewer for highlighting the importance of deep white matter in the neuropathology of HAND. We agree that the lack of dedicated deep white matter sampling is a limitation. In our study, CNS samples were obtained as bulk region-targeted tissue and may have variably included adjacent white matter, but deep white matter was not sampled as a distinct anatomical compartment. We have revised the methods and discussion (and Figure 4 as per next Reviewer comment) to make this explicit and to clarify that our findings reflect immune features within bulk tissue from targeted regions rather than tissue-specific neuropathology. Consequently, our study cannot directly address white matter–predominant mechanisms of injury. We would emphasize that the primary aim of this study was not to define classical neuropathological substrates of HAND, but to characterize region-resolved immune-genomic architecture across CNS compartments. Bulk tissue sampling still allows investigation of spatially heterogeneous immune signatures, although it limits compartment- and cell-specific interpretation. We agree that future studies incorporating standardized sampling of cortical gray matter, juxtacortical white matter, and deep white matter will be essential to better resolve the anatomical substrates of neuroimmune injury in treated HIV.

- Discussion, limitation section lines 583-593: “CNS regions were analyzed as bulk tissue without separation of gray and white matter compartments, and deep white matter was not systematically sampled as a distinct anatomical region. With the exception of TSC, all sites showed gray matter predominance. HIV-associated neurocognitive disorders have been linked to pathology in deep white matter and selected subcortical regions[79]. As such, our study cannot directly address white matter–predominant mechanisms of injury and precludes resolution of tissue-specific differences arising from distinct T-cell subpopulations and their local microenvironmental niches[80]. Instead, our findings should be interpreted as reflecting immune architecture within bulk region-targeted tissue, encompassing gray matter and immediately adjacent white matter, and does not allow distinction between cortical, juxtacortical, and deep white matter T cells.”
- Methods 640-644: “CNS samples were obtained as region-targeted bulk tissue, without separation of gray and white matter compartments. As such, samples included grey matter and variably adjacent white matter, but deep white matter was not systematically or independently sampled as a distinct anatomical compartment”

We also agree that the timing, onset, and trajectory of cognitive impairment are critical to interpreting the relationship between immune features and brain function and cannot be determined within the constraints of this cross-sectional design. We temperate language and removed terminology implying causality throughout the manuscript in this regard. We cannot determine whether the observed TCRR features preceded cognitive decline, contributed to it, or instead reflect ongoing immune surveillance or adaptation following prior CNS injury. As the reviewer notes, some of the observed deficits may represent remote or cumulative damage, with current immune signatures reflecting responses to past injury rather than active pathogenic processes. We have revised the discussion to more explicitly acknowledge this temporal ambiguity and to avoid causal interpretation, framing our findings as associations that are consistent with, but do not establish, a role for CNS T-cell architecture in neurocognitive outcomes, and alternative explanations, including immune responses to antecedent injury, remain plausible.

- Discussion, Limitations section 599-620: “Fourth, the cross-sectional design precludes determination of the temporal sequence between TCRR and cognition: we cannot establish whether the observed TCRR features preceded cognitive impairment, contributed to its development, or instead reflect persistent immune surveillance or adaptation following prior CNS injury. Thus, some of the observed associations may represent immune responses to historical or cumulative damage rather than drivers of ongoing pathology, or both. This limitation is partially mitigated by the nature of TCRR itself: it reflects cumulative antigenic exposure and long-term immune dynamics. Longitudinal studies in peripheral blood indicate that TCRR architecture evolves gradually over years to decades, largely driven by aging and chronic antigen exposure[81]. Over shorter timeframes (months to one year), dominant clonotypes and overall repertoire structure appear relatively stable[82], despite ongoing antigen-driven fluctuations or HIV infection[30,83]. Unlike soluble inflammatory markers, which can shift over hours to days, the clonal composition of T cell populations, especially in an immunologically privileged compartment like the CNS, reflects cumulative antigen exposure and clonal selection over months to years. Significant reshaping of the CNS TCR repertoire would require sustained antigenic drive or major immune reconstitution events, neither of which is expected in ART-suppressed individuals over short timeframes; however, longitudinal data in CNS tissues are lacking. In this context, although TCRR was measured at a single time point, its short-term stability in peripheral compartments, together with the limited change in cognitive status in our cohort, in line with prior longitudinal studies showing relative stability of cognitive trajectories over few years in PWH[84,85], support the plausibility of biologically meaningful associations within this timeframe, although causal relationships cannot be inferred”

This part of manuscript changes included also some done in accordance with Rev#2 comment on describing further data on the cognitive trajectory of the study population, that was added. Prior neurocognitive assessments were available for 8 participants, at a median time of 20 months (IQR 15; 33) before the assessment used in the present study. Across these participants, the median change in GDS was 0.28 (IQR −0.06; 0.53). Two participants transitioned from normal to impaired performance, whereas the remaining participants stayed within the same category, including two who improved GDS staying within the normal performance category, and four persistently impaired with worsening GDS. After normalizing for time between assessments, the median change in GDS was +0.01/month (IQR −0.004 to +0.02), corresponding to approximately +0.12/year. Thus, although some quantitative worsening occurred, global impairment status was largely stable over the available pre-autopsy interval (almost 2 years). This is in line with prior studies showing relatively stable cognitive trajectories over medium-short observation times in PWH (PMID: 17914066; 25362201).

**Part III – Minor Reviewer #3:** Because of the issues discussed above the figure showing the sites of tissue that were analysed is inaccurate.

**AR:** We agree that the original schematic could be interpreted as implying a higher degree of anatomical precision and compartment specificity than is supported by our sampling approach. Although tissue was consistently obtained from defined anatomical regions (e.g., hippocampus, thoracic spinal cord), samples represent region-targeted bulk tissue and may include variable contributions from parenchymal, perivascular, and meningeal/interface compartments, as acknowledged in the limitations section. To address this concern, we have revised Figure 4 to more accurately reflect the sampling context. Specifically, we added inset schematic panels illustrating representative neuroimmune interfaces in the hippocampus (including proximity to the choroid plexus) and spinal cord (including meningeal and CSF-associated compartments) and clarified in the legend that these regions may capture mixed anatomical and immune compartments rather than discrete, histologically defined structures. We have also revised the figure legend to explicitly state that immune signals detected in these regions likely reflect this region-dependent sampling of neuroimmune interfaces rather than precise anatomical localization, and we clarified that, although neuroimmune interfaces are present throughout the CNS, their relative contribution to bulk tissue sampling and immune signal detection is not uniform across regions, and is likely greater in areas such as the hippocampus and spinal cord compared with more immune-restricted regions. We believe these changes improve the accuracy of the schematic representation and better align the figure with the biological interpretation and limitations of the study.
