# Supplementary material for: Central Nervous System T-cell immune architecture, and not HIV burden, tracks with cognition under long-term viral suppression
Source: PLoS Pathog. 2026 Jun 15;22(6):e1014351. doi: 10.1371/journal.ppat.1014351 (PMC13286276; doi:10.1371/journal.ppat.1014351)
Supplement: Peer review history

# Peer review: Decision Letter 0

**DOI:** 10.1371/journal.ppat.1014351.r001

**Authors:** Jason Brenchley

16 Mar 2026

-->PPATHOGENS-D-26-00422

Central Nervous System T-cell immune architecture, and not HIV burden, tracks with cognition under long-term viral suppression

PLOS Pathogens

Dear Dr. Trunfio,

Thank you for submitting your manuscript to PLOS Pathogens. After careful consideration, we feel that it has merit but does not fully meet PLOS Pathogens's publication criteria as it currently stands. Therefore, we invite you to submit a revised version of the manuscript that addresses the points raised during the review process.

Please submit your revised manuscript by May 15 2026 11:59PM. If you will need more time than this to complete your revisions, please reply to this message or contact the journal office at plospathogens@plos.org. When you're ready to submit your revision, log on to https://www.editorialmanager.com/ppathogens/ and select the 'Submissions Needing Revision' folder to locate your manuscript file.

Please include the following items when submitting your revised manuscript:

* A letter that responds to each point raised by the editor and reviewer(s). You should upload this letter as a separate file labeled 'Response to Reviewers'. This file does not need to include responses to any formatting updates and technical items listed in the 'Journal Requirements' section below.

* A marked-up copy of your manuscript that highlights changes made to the original version. You should upload this as a separate file labeled 'Revised Manuscript with Track Changes'.

* An unmarked version of your revised paper without tracked changes. You should upload this as a separate file labeled 'Manuscript'.

If you would like to make changes to your financial disclosure, competing interests statement, or data availability statement, please make these updates within the submission form at the time of resubmission. Guidelines for resubmitting your figure files are available below the reviewer comments at the end of this letter.

We look forward to receiving your revised manuscript.

Kind regards,

Jason M. Brenchley

Academic Editor

PLOS Pathogens

Richard Koup

Section Editor

PLOS Pathogens

Sumita Bhaduri-McIntosh

Editor-in-Chief

PLOS Pathogens

orcid.org/0000-0003-2946-9497

Michael Malim

Editor-in-Chief

PLOS Pathogens

orcid.org/0000-0002-7699-2064

**Additional Editor Comments:**

The reviewers raised concerns that need to be addressed. If the authors can address the concerns, a revision could be considered.

**Journal Requirements:**

If the reviewer comments include a recommendation to cite specific previously published works, please review and evaluate these publications to determine whether they are relevant and should be cited. There is no requirement to cite these works unless the editor has indicated otherwise.

1) Please ensure that the CRediT author contributions listed for every co-author are completed accurately and in full.

At this stage, the following Authors/Authors require contributions: Mattia Trunfio, Gemma Caballero, Vanessa Gomez-Moreno, Simon A. Mallal, Celestine N. Wanjalla, Angela Jones, Karen Beeri, Alan Wells, Sarah LaMere, Ben Gouaux, Donald R. Franklin, Michael Corley, Ronald J. Ellis, David J. Moore, Scott L. Letendre, Davey Smith, Antoine Chaillon, and Sara Gianella. Please ensure that the full contributions of each author are acknowledged in the "Add/Edit/Remove Authors" section of our submission form.

The list of CRediT author contributions may be found here: https://journals.plos.org/plospathogens/s/authorship#loc-author-contributions

2) We do not publish any copyright or trademark symbols that usually accompany proprietary names, eg ©, ®, or TM (e.g. next to drug or reagent names). Therefore please remove all instances of trademark/copyright symbols throughout the text, including:

- ® on page: 21.

3) Please upload all main figures as separate Figure files in .tif or .eps format. For more information about how to convert and format your figure files please see our guidelines:

https://journals.plos.org/plospathogens/s/figures

4) Some material included in your submission may be copyrighted. According to PLOSu2019s copyright policy, authors who use figures or other material (e.g., graphics, clipart, maps) from another author or copyright holder must demonstrate or obtain permission to publish this material under the Creative Commons Attribution 4.0 International (CC BY 4.0) License used by PLOS journals. Please closely review the details of PLOSu2019s copyright requirements here: PLOS Licenses and Copyright. If you need to request permissions from a copyright holder, you may use PLOS's Copyright Content Permission form.

Please respond directly to this email and provide any known details concerning your material's license terms and permissions required for reuse, even if you have not yet obtained copyright permissions or are unsure of your material's copyright compatibility. Once you have responded and addressed all other outstanding technical requirements, you may resubmit your manuscript within Editorial Manager.

Potential Copyright Issues:

i) Figure 4. Please confirm whether you drew the images / clip-art within the figure panels by hand. If you did not draw the images, please provide (a) a link to the source of the images or icons and their license / terms of use; or (b) written permission from the copyright holder to publish the images or icons under our CC BY 4.0 license. Alternatively, you may replace the images with open source alternatives. See these open source resources you may use to replace images / clip-art:

- https://commons.wikimedia.org

- https://openclipart.org/.

5) In the online submission form, you indicated that De-identified data are available to qualified researchers upon reasonable request and subject to approval by the University of California, San Diego Human Research Protections Program (IRB#160563, IRB#171024) and execution of an appropriate data use agreement. Requests may be directed to the corresponding authors.. All PLOS journals now require all data underlying the findings described in their manuscript to be freely available to other researchers, either

1. In a public repository

2. Within the manuscript itself

3. Uploaded as supplementary information.

This policy applies to all data except where public deposition would breach compliance with the protocol approved by your research ethics board. If your data cannot be made publicly available for ethical or legal reasons (e.g., public availability would compromise patient privacy), please explain your reasons by return email and your exemption request will be escalated to the editor for approval. Your exemption request will be handled independently and will not hold up the peer review process, but will need to be resolved should your manuscript be accepted for publication. One of the Editorial team will then be in touch if there are any issues

**Reviewers' Comments:**

Reviewer's Responses to Questions

**Part I - Summary**

Please use this section to discuss strengths/weaknesses of study, novelty/significance, general execution and scholarship.

Reviewer #1: Trunfio and colleagues examined how HIV-1 DNA and TCR repertoires across distinct CNS regions relate to cognitive impairment in post-mortem tissues from ART-suppressed PWH, with cognitive scores assessed within a year of death. Using multivariate regression, they found that TCR diversity was highest in blood and largely compartmentalized across CNS anatomical sites. Notably, greater TCR diversity in the hippocampus and thoracic spinal cord associated with worse verbal and motor function, and regional HIV-specific clonotype frequencies also correlated with cognitive outcomes. There were no differences in HIV-1 DNA between sites (excluding the blood) nor associations to cognitive scores. Strengths of the study include the unique and comprehensive sampling opportunity of the Last Gift Cohort. All subjects had sustained ART suppression, which is a strength relative to similar studies that have been limited by cohort heterogeneity in viremic status. The novelty in this study is that to my knowledge, TCR repertoires across distinct regions of the brain in PWH have not been characterized in PWH. The major driving weaknesses are that it is devoid of inflammatory measurements, either soluble in the plasma or CNS or by transcriptomics. This is important because most of these patients fit the definition of immune non-responders (CD4<350) which are well known to exhibit persistently high levels of inflammation. A second weakness is that the associations between TCRRs and specific cognitive scores are not necessarily found within the brain regions that govern those neurological functions, leaving the implications unclear. The authors do touch on this caveat in the discussion in sampling TCR-high parenchymal versus TCR-low tissue subregions, however the authors would need spatial profiling to confirm this.

Reviewer #2: Trunfio et al. compared metrics of HIV viral persistence and T cell receptor repertoire (TCRR) in multiple central nervous system (CNS) sites to cognitive measurements prior to death in 12 patients. While HIV metrics did not correlate with cognitive performance, some TCRR metrics correlated with some cognitive measures, with generally more richness/diversity tracking with worse cognitive scores.

Overall, the article is well-written and figures are clear.

Even under highly effective anti-retroviral therapy, cogitative pathology still occurs. This study seeks to determine if there are links between infection, T cell receptor repertoire, and cognitive performance.

A particular strength is the samples assessed as CNS tissue from people with HIV is relatively rare.

Reviewer #3: This is an exhaustive and comprehensive study exploring HAND pathogenesis in the context of ART using precious and very rare autopsy samples from the Last Gift Program. The authors have used state of the art methods and analyses. The weaknesses are detailed in the manuscript including the small number of brains but two exceptionally important issues are not discussed:

* the tissues that were analysed did not include deep white matter. The methods used bulk tissue with the authors explaining that there would be some adjacent tissue ie white matter but the precise neuropathology of HAND is not understood. Certainly in the past it was more of a deep white matter pathology involving especially the frontal white matter and part of the basal ganglia but not all of the basal ganglia. It would have been far better to have included both cortical tissue and deep white matter.

*the second issue relates to the cognitive impairment - it is not at all clear when this developed: we know when it was tested for but not its onset and progression. This is fundamental to understanding pathogenesis as at least some of the deficits likely reflected past damage with an appropriate current TCRR response now that has controlled the limited replication of whole virus or part thereof. This then changes the whole new suggested paradigm of pathogenesis and makes it only a possibility rather than a certainty.

**********

**Part II – Major Issues: Key Experiments Required for Acceptance**

Please use this section to detail the key new experiments or modifications of existing experiments that should be absolutely required to validate study conclusions.

Generally, there should be no more than 3 such required experiments or major modifications for a "Major Revision" recommendation. If more than 3 experiments are necessary to validate the study conclusions, then you are encouraged to recommend "Reject".

Reviewer #1: - The authors find associations between regional T cell receptor repertoires and worse outcomes in specific neurocognitive measures such as verbal and motor functions (Figure 4); however, the significance of these findings is unclear because the specific regions in which these associations are observed — the hippocampus and thoracic spinal cord — do not primarily govern these functions. The hippocampus is mostly involved with memory, and the thoracic spinal cord primarily controls trunk muscle movement, not verbal or motor neurocognitive performance.

- Similarly, the authors observed CMV-specific clonotypes in the TSC to correlate with worse learning scores, however the TSC does not control learning.

- Do the authors have CD8 T cell counts as well? Many of these subjects likely have inverted CD4/CD8 ratios, and it would be helpful to know whether TCRs are more reflective of CD4 versus CD8 repertoires.

- The association between higher TCR diversity and worse neurocognitive outcomes is somewhat contrary to the broader literature. Oligoclonality or skewed repertoire diversity — rather than expanded diversity — has been associated with neurocognitive impairment. The significance of this finding and the potential mechanism underlying it is somewhat uncertain.

- The positive association between HIV-specific T cell clonotype frequency and better GDS and processing speed scores is intriguing but difficult to interpret. One possibility is that a higher proportion of HIV-specific clonotypes reflects more active immune surveillance of the tissue. However, neither the HIV-specific clonotype frequency nor the neurocognitive outcomes correlated with HIV DNA levels in these regions, leaving the biological significance of this association uncertain.

- Lines 395-397 regarding the underlying nature of the high TCR richness. I do agree that leukocytosis in the CNS could plausibly explain the findings. It is unfortunate that the authors do not have the cellular or transcriptomic data to test this hypothesis.

Reviewer #2: 1: A key aspect of the study is correlating measures of viral persistence and T cell receptor repertoire with cognitive performance scores. There will necessarily be a gap of time between biological measurements performed after the patient’s death and cognitive assessment prior to death. How stable were the cognitive metrics over time in this cohort? Alternatively, is there data on expected progression in a similar cohort? Was the length of time between biological measurements and cognitive assessment considered in any of the modeling?

2: Similarly, by necessity sampling of many CNS sites cannot be performed longitudinally and must be a snapshot of a particular time. How much variation in T cell receptor repertoire metrics is seen in sites that can be sampled over time (eg. PBMCs, cerebrospinal fluid) either in this cohort or in other published work, even from animal models if necessary?

In both cases, any additional data or references could strengthen the paper.

Reviewer #3: Please see above - white matter needs to be obtained and more precise details for the cognitive impairment

**********

**Part III – Minor Issues: Editorial and Data Presentation Modifications**

Please use this section for editorial suggestions as well as relatively minor modifications of existing data that would enhance clarity.

Reviewer #1: - Minor: it would improve readability if the acronyms were also spelled out in the text, when they are first referred to.

- Minor: lines 207-212, the authors should refer to the specific figure legend in the text.

Reviewer #2: 1: Though this is an acknowledged limitation of the study, a biological mechanism for the observed correlations is lacking. Some method of assessing inflammation could significantly improve the link.

2: Do the multiple measures of the HIV reservoir correlate with each other in each tissue? For example, is more HIV DNA associated with more 2LTR, usGag, or msTat/Rev? If not, (for example a lot of DNA but not ms transcripts) does that suggest something about the amount of active viral replication in the particular tissue?

3: Line 121: “emerged as an important contributor…” How accurate is the term contributor vs. correlate. Is there direct evidence of contribution in all cited cases?

4: Line 140 cites perturbed and abnormal TCRR in PWH in peripheral blood. Is that the case in this study if the peripheral blood TCRR were compared to uninfected controls?

5: Line 176. First use of abbreviations in the results eg. FMC, HPC, usGAG, etc. should be defined. Figure legends do a good job of this, though.

6: Figure 5 radial diagrams. The % labels are hard to read, could use larger and/or darker font.

7: Line 504-505 Sentence beginning “Although substantial inter-host variability,” is lacking a word. Maybe, “Although there was substantial…”

8: Methods 3.4 for the TCR sequencing could use more detail on the IMMUNOVerse platform.

Reviewer #3: Because of the issues discussed above the figure showing the sites of tissue that were analysed is inaccurate.

**********

PLOS authors have the option to publish the peer review history of their article (what does this mean?). If published, this will include your full peer review and any attached files.

If you choose “no”, your identity will remain anonymous but your review may still be made public.

**Do you want your identity to be public for this peer review?**  For information about this choice, including consent withdrawal, please see our Privacy Policy.

Reviewer #1: No

Reviewer #2: No

Reviewer #3: No

[NOTE: If reviewer comments were submitted as an attachment file, they will be attached to this email and accessible via the submission site. Please log into your account, locate the manuscript record, and check for the action link "View Attachments". If this link does not appear, there are no attachment files.]

**Figure resubmission:**

-->While revising your submission, we strongly recommend that you use PLOS’s NAAS tool (https://ngplosjournals.pagemajik.ai/artanalysis) to test your figure files. NAAS can convert your figure files to the TIFF file type and meet basic requirements (such as print size, resolution), or provide you with a report on issues that do not meet our requirements and that NAAS cannot fix.

After uploading your figures to PLOS’s NAAS tool - https://ngplosjournals.pagemajik.ai/artanalysis, NAAS will process the files provided and display the results in the "Uploaded Files" section of the page as the processing is complete. If the uploaded figures meet our requirements (or NAAS is able to fix the files to meet our requirements), the figure will be marked as "fixed" above. If NAAS is unable to fix the files, a red "failed" label will appear above. When NAAS has confirmed that the figure files meet our requirements, please download the file via the download option, and include these NAAS processed figure files when submitting your revised manuscript.

**Reproducibility:**

To enhance the reproducibility of your results, we recommend that authors of applicable studies deposit laboratory protocols in protocols.io, where a protocol can be assigned its own identifier (DOI) such that it can be cited independently in the future. Additionally, PLOS ONE offers an option to publish peer-reviewed clinical study protocols. Read more information on sharing protocols at https://plos.org/protocols?utm_medium=editorial-email&utm_source=authorletters&utm_campaign=protocols

Attachment Submitted filename: PPATHOGENS-D-26-00422_reviewer comments.docx

# Author comment: Author response to Decision Letter 1

**DOI:** 10.1371/journal.ppat.1014351.r002

13 May 2026

Attachment Submitted filename: Response To Reviewers.docx

# Peer review: Decision Letter 1

**DOI:** 10.1371/journal.ppat.1014351.r003

**Authors:** Jason Brenchley

4 Jun 2026

Dear Dr. Trunfio,

We are pleased to inform you that your manuscript 'Central Nervous System T-cell immune architecture, and not HIV burden, tracks with cognition under long-term viral suppression' has been provisionally accepted for publication in PLOS Pathogens.

Before your manuscript can be formally accepted you will need to complete some formatting changes, which you will receive in a follow up email. A member of our team will be in touch with a set of requests.

Please note that your manuscript will not be scheduled for publication until you have made the required changes, so a swift response is appreciated.

IMPORTANT: The editorial review process is now complete. PLOS will only permit corrections to spelling, formatting or significant scientific errors from this point onwards. Requests for major changes, or any which affect the scientific understanding of your work, will cause delays to the publication date of your manuscript.

Should you, your institution's press office or the journal office choose to press release your paper, you will automatically be opted out of early publication. We ask that you notify us now if you or your institution is planning to press release the article. All press must be co-ordinated with PLOS.

Thank you again for supporting Open Access publishing; we are looking forward to publishing your work in PLOS Pathogens.

Best regards,

Jason M. Brenchley

Academic Editor

PLOS Pathogens

Richard Koup

Section Editor

PLOS Pathogens

Sumita Bhaduri-McIntosh

Editor-in-Chief

PLOS Pathogens

orcid.org/0000-0003-2946-9497

Michael Malim

Editor-in-Chief

PLOS Pathogens

orcid.org/0000-0002-7699-2064

***********************************************************

The reviewers believe their concerns have been addressed.

Reviewer Comments (if any, and for reference):

Reviewer's Responses to Questions

**Part I - Summary**

Please use this section to discuss strengths/weaknesses of study, novelty/significance, general execution and scholarship.

Reviewer #2: Trunfio et al. compared metrics of HIV viral persistence and T cell receptor repertoire (TCRR) in multiple central nervous system (CNS) sites to cognitive measurements prior to death in 12 patients. While HIV metrics did not correlate with cognitive performance, some TCRR metrics correlated with some cognitive measures, with generally more richness/diversity tracking with worse cognitive scores.

Overall, the article is well-written and figures are clear.

Even under highly effective anti-retroviral therapy, cogitative pathology still occurs. This study seeks to determine if there are links between infection, T cell receptor repertoire, and cognitive performance.

Reviewer #3: The authors ahve adequately addressed the issues raised

**********

**Part II – Major Issues: Key Experiments Required for Acceptance**

Please use this section to detail the key new experiments or modifications of existing experiments that should be absolutely required to validate study conclusions.

Generally, there should be no more than 3 such required experiments or major modifications for a "Major Revision" recommendation. If more than 3 experiments are necessary to validate the study conclusions, then you are encouraged to recommend "Reject".

Reviewer #2: The authors have sufficiently addressed the potential major issues by adding to the text.

Reviewer #3: Not applicable

**********

**Part III – Minor Issues: Editorial and Data Presentation Modifications**

Please use this section for editorial suggestions as well as relatively minor modifications of existing data that would enhance clarity.

Reviewer #2: The authors have sufficiently addressed the minor issues.

Reviewer #3: Not applicable

**********

PLOS authors have the option to publish the peer review history of their article (what does this mean?). If published, this will include your full peer review and any attached files.

If you choose “no”, your identity will remain anonymous but your review may still be made public.

**Do you want your identity to be public for this peer review?**  For information about this choice, including consent withdrawal, please see our Privacy Policy.

Reviewer #2: No

Reviewer #3: No

# Editor report: Acceptance letter

**DOI:** 10.1371/journal.ppat.1014351.r004

**Authors:** Jason Brenchley

Dear Dr. Trunfio,

We are delighted to inform you that your manuscript, "Central Nervous System T-cell immune architecture, and not HIV burden, tracks with cognition under long-term viral suppression," has been formally accepted for publication in PLOS Pathogens.

We have now passed your article onto the PLOS Production Department who will complete the rest of the pre-publication process. All authors will receive a confirmation email upon publication.

The corresponding author will soon be receiving a typeset proof for review, to ensure errors have not been introduced during production. Please review the PDF proof of your manuscript carefully, as this is the last chance to correct any scientific or type-setting errors. Please note that major changes, or those which affect the scientific understanding of the work, will likely cause delays to the publication date of your manuscript. Note: Proofs for Front Matter articles (Pearls, Reviews, Opinions, etc...) are generated on a different schedule and may not be made available as quickly.

Soon after your final files are uploaded, the early version of your manuscript, if you opted to have an early version of your article, will be published online. The date of the early version will be your article's publication date. The final article will be published to the same URL, and all versions of the paper will be accessible to readers.

For Research Articles, you will receive an invoice from PLOS for your publication fee after your manuscript has reached the completed accept phase. If you receive an email requesting payment before acceptance or for any other service, this may be a phishing scheme. Learn how to identify phishing emails and protect your accounts at https://explore.plos.org/phishing.

Thank you again for supporting open-access publishing; we are looking forward to publishing your work in PLOS Pathogens.

Best regards,

Sumita Bhaduri-McIntosh

Editor-in-Chief

PLOS Pathogens

orcid.org/0000-0003-2946-9497

Michael Malim

Editor-in-Chief

PLOS Pathogens

orcid.org/0000-0002-7699-2064
